# PD-L2 of tumor-derived exosomes mediates the immune escape of cancer cells via the impaired T cell function

**DOI:** 10.1038/s41419-024-07191-7

**Published:** 2024-11-07

**Authors:** Tongfeng Liu, Shuwen Cheng, Bo Peng, Haojing Zang, Xiaofeng Zhu, Xuetong Wang, Xujie Zhao, Yinmin Gu, Yongbo Pan, Hongbo Hu, Shan Gao

**Affiliations:** 1 Medical College, Guizhou University, Guiyang, China; 2 Zhongda Hospital, School of Life Sciences and Technology, Advanced Institute for Life and Health, Southeast University, Nanjing, China; 3 Medical School of Nanjing University, Nanjing, China; 4 School of Biomedical Engineering (Suzhou), Division of Life Sciences and Medicine, University of Science and Technology of China, Hefei, China; 5 Department of Microbiology and Immunology, Shanxi Medical University, Taiyuan, Shanxi China; 6 Guangdong Cardiovascular Institute, Guangdong Provincial People’s Hospital, Guangdong Academy of Medical Sciences, Taiyuan, China; 7 Center for Immunology and Hematology, Department of Biotherapy and Cancer Center, State Key Laboratory of Biotherapy, West China Hospital, Sichuan University, Chengdu, China

**Keywords:** Cancer microenvironment, Tumour immunology

## Abstract

The function of PD-1/PD-L1 axis have been intensively studied for immune escape of various cancers. However, the underlying function of PD-L2 remains poorly understood. Here, we demonstrate that PD-L2 is majorly expressed in exosomes with surface localization by clear cell renal cell carcinoma (ccRCC) cells. Tumor cell-derived exosome PD-L2 (TDE-PD-L2) exhibits high expression compared with TDE-PD-L1 in various cancers. In the absence of adaptive immune, TDE-PD-L2 suppresses tumor growth and metastasis. Under immune competence condition, TDE-PD-L2 is hijacked by immune cells in a PD-1-dependent manner to systematically dampen function of T cells via the increased proportion of the regulatory T cells and the decreased proportion of cytotoxic CD8^+^ T cells in both tumor-infiltrating T cells and spleen. The effects of TDE-PD-L2 on tumor is restored by antibodies targeting PD-L2. Collectively, we demonstrate that PD-1/TDE-PD-L2 axis systematically suppresses T cell functions, representing a potentially therapeutic strategy for ccRCC treatment.

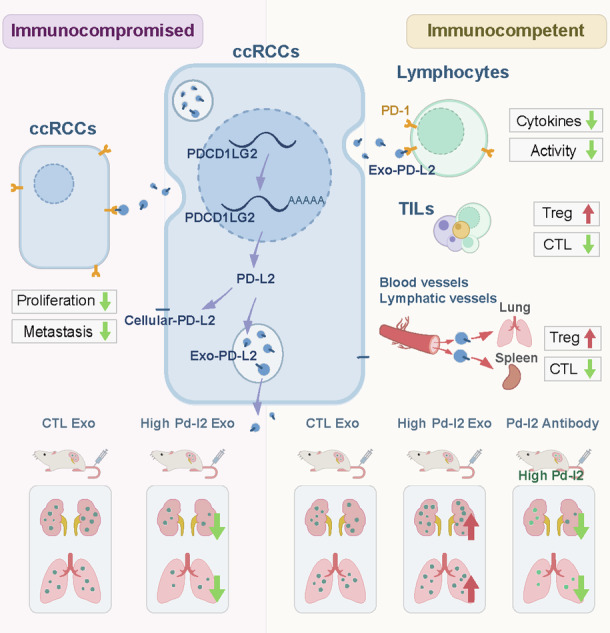

## Introduction

Immune checkpoints play a vital role in mediating the escape of tumor cells for immunosurveillance [1]. The programmed cell death 1 (PD-1) receptor is one of the crucial immune checkpoint molecules which dampens immune responses via engagements with its two ligands, PD-1 ligand 1 (PD-L1) and PD-L2 expressed by cancer cells [2]. Especially PD-1/PD-L1 axis has been widely studied in various cancer types, and found to promote the immune escape of tumor cells [3, 4]. Targeting this axis has also demonstrated some benefits for several cancer patients [5]. However, the potential function of PD-L2 in immune escape of tumor cells has been poorly studied. Moreover, the expression regulation of *PDCD1*/*PDCD1LG1* that respectively encodes PD-1/PD-L1 has been studied in multiple levels including transcription, translation, post-transcription, and post-translation etc. [6–8]. The regulation of *PDCD1LG2* encoding PD-L2 expression is incompletely understood.

Extracellular vesicles (EVs) are broadly classified into exosomes and microvesicles (MVs) based on their different size, as important mediators of extracellular communication to regulate a diverse range of biological processes, including the immune system [9, 10]. Exosome is considered 30-200 nm in diameter, while MV is suggested as 200–1000 nm in diameter. Exosomes derived from tumor cells (TDEs) are essential for regulation of various cancer progression, including metastasis and immune escape [9, 11]. Whether PD-L2 is carried by TDEs to mediate immune escape remains elusive.

In this study, we demonstrate that renal clear cell carcinoma (ccRCC) cells secrete PD-L2 via exosomes, which promotes growth and metastasis of ccRCC tumors via the increased regulatory T (Treg) cells and the decreased cytotoxic CD8+ T (CTL) cells.

## Materials and methods

### Cell lines, clinical specimens, and mice

786-O, 769-P, ACHN and Caki-1 cells were maintained in RPMI1640 medium (Gibco, Cat#11875119) supplemented with 10% FBS (Gibco, Cat#10099158). HK2 cells were maintained in DMEM/F-12 medium (Gibco, Cat#11330032) containing 10% FBS. Renca cells were maintained in RPMI1640 medium supplemented with 10% FBS, 1% Sodium Pyruvate (Gibco, Cat#11360070) and 1 × MEM Non-Essential Amino Acids Solution (NEAA) (Gibco, Cat#11140035). The human embryonic kidney HEK293T cells were cultured in DMEM (Gibco, Cat#11965092) supplemented with 10% FBS. These cell lines except Renca were purchased from the Shanghai Cell Bank Type Culture Collection Committee (Shanghai, China), and verified by short tandem repeat assays for their identification. Renca cell line was from American Type Culture Collection. The PD-1 KO Jurkat cell line used in this study was constructed in our previous study [12]. Primary tumor cells separated from ccRCC tissues were cultured in RPMI1640 medium supplemented with 10% exosome-depleted FBS (Gibco, Cat#A27208-03), 1% Sodium Pyruvate and 1×MEM NEAA to collect exosomes. These cells were cultured at 37 °C in a humidified incubator with 5% CO^2^ and regularly tested negative for Mycoplasma contamination.

The pathologically diagnosed tissues (KIRC *n* = 9, COAD *n* = 3, PRAD *n* = 6, LUAD *n* = 4) were obtained from ZhongDa Hospital Southeast University in Nanjing, China. Written informed consent was obtained from the patients, and the study was approved by the Medical Ethics Committees of ZhongDa Hospital Southeast University (Nanjing, China).

Tissue arrays containing ccRCC and adjacent tissues were purchased from Shanghai Outdo Biotech CO in Shanghai, China. The company provided all clinical information of the patients with ccRCC. The tissue arrays used were listed in Table S1.

Male wild type (WT) and NOD-SCID (severe combined immune deficiency, SCID) BALB/c mice, aged 6–8 weeks, were obtained from Vital River Laboratory Animal Technology Co (Beijing, China). The mice were housed in a specific pathogen-free (SPF) animal facility with a temperature of 28 °C and humidity of 50%. The use of animals in this study was approved by the Ethics Committee for the Use of Experimental Animals of the Suzhou Institute of Biomedical Engineering and Technology, Chinese Academy of Sciences (Suzhou, Jiangsu, China).

### Plasmids and antibodies

To generate expression plasmids for Human PD-L2 and Mouse Pd-l2, the coding sequence (CDS) of these genes was cloned into the pLVX-IRES-Neo vector, with a flag-tag at the C-terminus.

To create knockdown (KD) cell lines targeting specific genes, short hairpin RNAs (shRNAs) targeting Rab27a were cloned into the pSIH1-H1-Puro vector. The specific sequences of the shRNAs used for KD are listed in Table S2.

Reagents and antibodies used are listed in Table S1.

### Statistical analysis

Statistical analysis was performed using GraphPad Prism 6 software. Data of bar graphs represents as fold change or percentage relative to control with SD of three independent experiments. Normally distributed data were analyzed using Student’s t test. Two-way ANOVA was used for the cell proliferation assay. Statistical significance was defined as p < 0.05. Levels of significance were indicated as *p < 0.05, **p < 0.01, ***p < 0.001, and ****p < 0.0001.

Additional methods can be found in the Supplementary Materials and Methods in Supplementary information.

## Results

### PD-L2 is expressed on the exosome surface

**Fig. 1:**
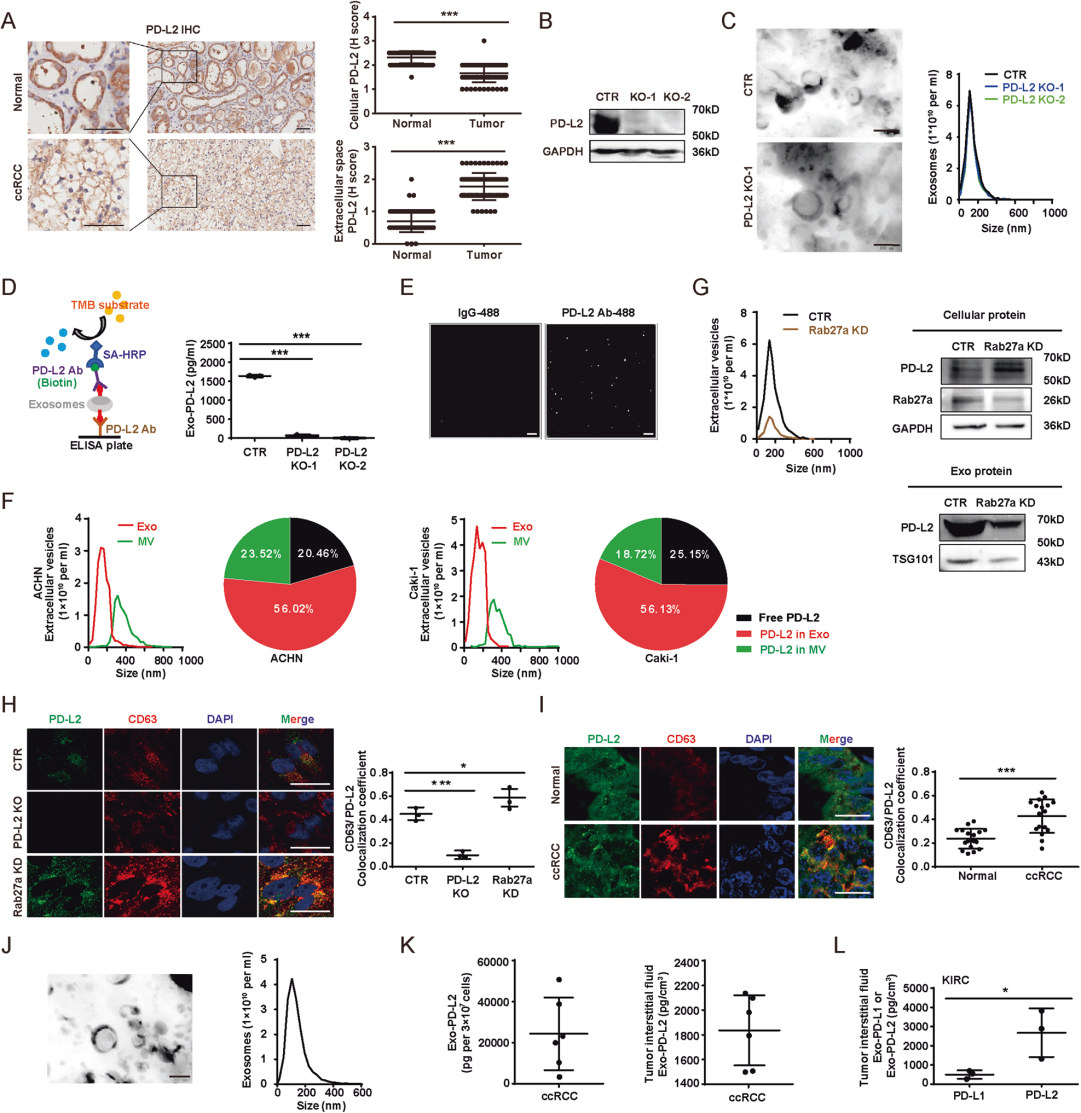
PD-L2 is expressed on exosome surface. **A** Representative stained tissue microarray cores of PD-L2 (left). Quantification of cellular and extracellular PD-L2 in the ccRCC tissues versus the matched adjacent tissues (*n* = 75 pairs) (right). Scale bars, 20 µm. **B** Immunoblot analysis for the KO efficiency of PD-L2 in 786-O cells. **C** Exosomes purified from control (CTR) or PD-L2 KO 786-O cells and subjected to be scanned by TEM (left) and NTA (right) to characterize the morphology, number and size distribution. Scale bars, 100 nm. **D** Schematic of ELISA design (left) and ELISA for surface PD-L2 of exosomes isolated from CTR and PD-L2 KO 786-O cells (right) (*n* = 3). **E** Representative images of exosomes isolated from PD-L2 OE 786-O cells, stained with either IgG-AF488 or PD-L2-AF488 antibodies, and captured under 488 fluorescent field of NTA. Scale bars, 500 nm. **F** Representative NTA images of both exosomes and MVs purified, and pie charts showing the relative proportion of extracellular PD-L2 proteins, including free parts, exosomes and MVs from ACHN (left) and Caki-1 (right) cells. **G** Representative NTA images of Rab27a KD effects on exosome number (upper left). Immunoblot analysis for cellular (upper right) or exosomal levels (bottom right) of the indicated proteins in CTR and Rab27a KD 786-O cells. **H** Representative images (left) and quantified colocalization (*n* = 3) (right) of PD-L2 (green) and CD63 (red) expression (right) from CTR, PD-L2 KO and Rab27a KD 786-O cells. The nuclei stained with DAPI (blue). Scale bars, 10 µm. **I** Representative images (right) and quantified colocalization coefficient of PD-L2 (green) and CD63 (red) expression in ccRCC tissues and the matched adjacent tissues on tissue microarray (*n* = 18 pairs) (left). Scale bars, 20 µm. **J** The characterization of exosomes isolated from primary tumor cells derived from ccRCC patients using TEM (left) and NTA (right). Scale bars, 100 nm. **K** ELISA analysis for exosomal PD-L2 from primary tumor cells (left) or tumor interstitial fluid (right) from ccRCC patients (*n* = 6). **L** ELISA analysis for PD-L2 and PD-L1 for exosomes isolated from primary tumor cells derived from ccRCC patients (*n* = 3). Data presented as means ± SD.*p < 0.05, ***p < 0.001, unpaired t test.

To explore whether PD-L2 plays an important role in immunosuppression of cancer, we examined the expression levels of both *PDCD1LG1* and *PDCD1LG2* from The Cancer Genome Atlas (TCGA), and found that *PDCD1LG2* exhibited higher expression levels than *PDCD1LG1* in multiple types of cancers including ccRCC (Fig. S1A), a major type of RCC [13, 14]. Moreover, the Human Protein Atlas database illustrated that PD-L2 was expressed in most cancer types (Fig. S1B). High expression of *PDCDLG2* was associated with higher pathologic stages of ccRCC based on TCGA data (Fig. S1C). We next examined PD-L2 expression using immunohistochemistry (IHC) in ccRCC tissue array, and found that PD-L2 expression pattern was unexpectedly changed with the higher extracellular expression in cancer tissues, in contrast to the higher cellular localization of PD-L2 in the adjacent tissues (Fig. 1A), indicating extracellular PD-L2 may regulates progression of ccRCC. It has been established that extracellular space constitutes a complex with fluid, matrix, and EVs etc. [15]. We thereby hypothesized that PD-L2 is secreted by ccRCC cells via EVs. We first generated two independent PD-L2 knockout (KO) cell lines using CRISPR-Cas9 technology (Fig. S1D), which is further verified by immunoblot (Fig. 1B). There was no obvious difference in exosome secretion and morphology between wildtype (WT) and KO cells by transmission electron microscopy (TEM) and nanoparticle tracking analysis (NTA) (Fig. 1C). Then we used an Enzyme-Linked Immunosorbent Assay (ELISA) to detect the contents of TDE-PD-L2, and found that the expression of PD-L2 on the surface of exosomes was derived from WT cells but not PD-L2 KO cells (Fig. 1D). Furthermore, immunofluorescence (IHF) staining against PD-L2 under the fluorescence field of NTA confirmed the expression of PD-L2 on the exosome surface (Fig. 1E). We used ultrafiltration combined with iodixanol density gradient centrifugation and further confirmed that PD-L2 was majorly associated with the fraction of exosomes in contrast to free fraction and MV fraction (Fig. 1F). It has been proved that both IFN-γ and IL-4 can effectively upregulate the expression of PD-L2 in either immune cells or cancer cells [16–18], so we further examined the effects of both IFN-γ and IL-4 on PD-L2 secretion of tumor cells. Indeed, the expressions of both cellular and exosomal PD-L2 in cancer cells were upregulated by INF-γ and IL-4 (Fig. S1E). All these data indicate that TDE-PD-L2 has the same membrane orientation as cell surface PD-L2, with its extracellular domain exposed on the outer surface of the exosome.

Exosomes are generated and released through a defined intracellular trafficking route [19]. Knockdown (KD) of Rab27a, a key component in exosomal biogenesis [20], resulted in less secretion of exosomes and subsequently increased cellular PD-L2 levels but decreased PD-L2 secretion (Fig. 1G). Given that CD63 is a hallmark protein for exosomes [21], we examined colocalization between PD-L2 and CD63, and found that the colocalization was higher in WT cells than in PD-L2 KO cells, while colocalization was lower in WT cells than in Rab27a KD cells (Fig. 1H), supporting that PD-L2 is secreted via exosomes in ccRCC cells. We also examined a panel of ccRCC cell lines and found that all of them expressed PD-L2 (Fig. S1F) and secreted exosomes (Fig. S1G, H). The ELISA analysis further revealed that TDE-PD-L2 level was significantly higher in exosomes of these ccRCC lines compared with the immortalized human HK2 tubular epithelial cell line, in which TDE-PD-L2 was undetectable (Fig. S1H). TDE-PD-L2 levels from the majority of ccRCC lines were higher in contrast to surface levels of the corresponding paired cell lines except 786-O (Fig. S1H). We further explored the PD-L2 expression in clinical samples. First, IHF results showed that PD-L2 exhibited higher colocalization with CD63 in cancer tissues than in paired adjacent tissues, indicating that PD-L2 is secreted by cancer cells (Fig. 1I). Next, the exosomes were verified by both TEM and NTA from both primary tumor cells and interstitial fluids from ccRCC patients (Fig. 1J and Fig. S1I). ELISA showed that PD-L2 was expressed in exosomes from both the isolated primary ccRCC patient-derived tumor cells and interstitial fluids of ccRCC patient tissues (Fig.1K). Moreover, PD-L2 exhibited significantly higher expression than PD-L1 in exosomes from both patient-derived primary tumor cells and different types of cancer patients, including ccRCC, colon, prostate, and lung adenocarcinoma (Figs. 1L and S1J). We performed ELISA to measure the expression of PD-L1 in exosomes of PD-L2 KO or overexpressing (OE) cells. The results indicated that the level of PD-L1 in exosomes increased following PD-L2 KO, while it decreased with PD-L2 OE (Fig. S1K), suggesting a negative feedback regulation relationship. Altogether, these data suggest that PD-L2 is expressed on the surface of either exosomes from cancer cell lines or primary tumor cells of ccRCC patients.

### TDE-PD-L2 is a tumor suppressor in an immunocompromised condition

To explore the potential effects of TDE-PD-L2 on tumor, we first examined the effects of PD-L2 on ccRCC cells in an immunocompromised condition. PD-L2 KO cells showed increased proliferation, invasion and migration compared to WT cells (Fig. S2A). On the contrary, overexpression (OE) of PD-L2 led to the opposite effects (Fig. S2B), indicating that cellular PD-L2 is a tumor suppressor. Moreover, tumor cells fed with exosomes from either PD-L2 KO cells or PD-L2 OE cells in comparison with tumor cells fed with exosomes of control cells had the similar effects on proliferation, invasion and migration (Fig. S2C, D), as the function of cellular PD-L2. These observations were consistent with our previous study and other studies [22–24], which showed that PD-1/PD-L1 axis is a tumor suppressor in tumor cells with an immunocompromised condition. Next, we wondered whether exogenously introduced TDE-Pd-l2 could suppress tumor growth and metastasis in vivo. We designed an orthotopic ccRCC transplantation model using a murine Renca cell line, and transplanted Renca cells in the left kidney of syngeneic mice as well as followed by tail vein injections of in vitro collected exosomes from either control or Pd-l2 OE counterparts (Fig. S2E). Clearly, TDE-Pd-l2 suppressed tumor growth and decreased the metastatic numbers and area as well as the weights of lung (Fig. S2F–H), indicating that TDE-Pd-l2 plays tumor suppressive roles in vivo. IHC revealed that group with Pd-l2 OE exosomes exhibited the increased levels of Pd-l2 than control group in tumor, lungs, and spleens, indicating that exosomes carrying Pd-l2 efficiently enter those organs (Fig.S2I). Overall, these data demonstrate that TDE-PD-L2 has an antitumor function in the absence of adaptive immunity.

### TDE-PD-L2 inhibits the function of T cells mainly via PD-1 in vitro

**Fig. 2:**
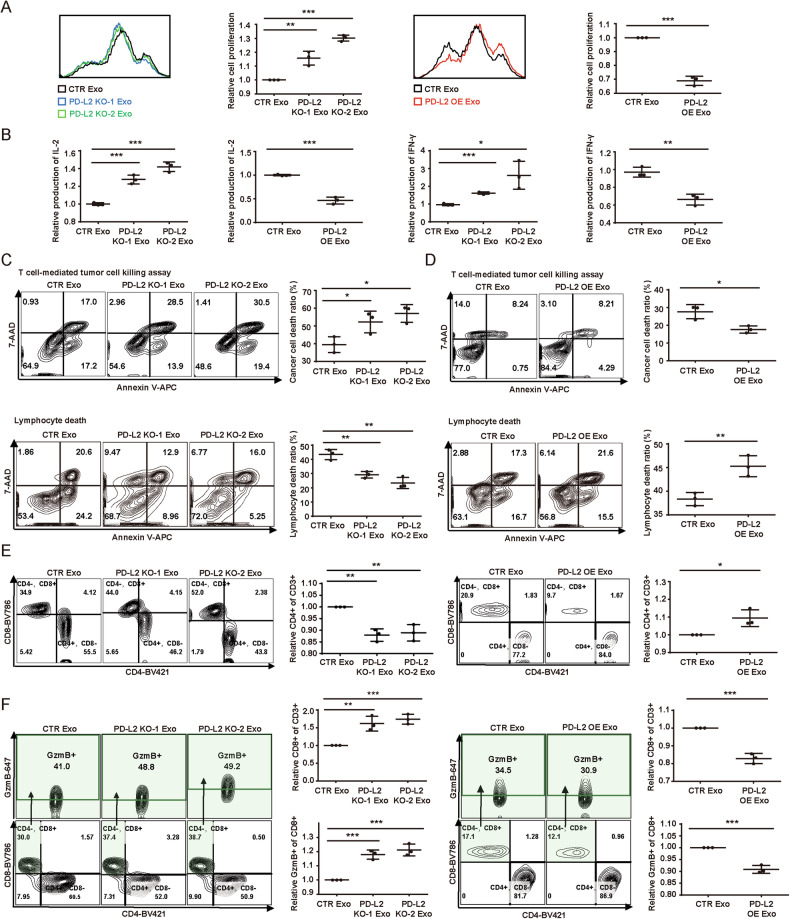
TDE-PD-L2 inhibits the activity and function of lymphocytes. **A** Representative FACS and quantification of lymphocyte proliferation as 1/mean fluoresce intensity (MFI) with the indicated exosomes. **B** Quantification of IL-2 (left) and IFN-γ (right) levels in the lymphocytes treated with the indicated exosomes. Representative FACS and quantification of death for cancer cells (top) and lymphocytes (bottom) fed with the CTR and PD-L2 KO (**C**) or PD-L2 OE (**D**) exosomes. **E** Representative FACS and quantification of CD4+ (top) and CD8+ (bottom) on CD3+ lymphocytes fed with the indicated exosomes. **F** Representative FACS and quantification of CD8^+^ lymphocytes expressing GzmB fed with the indicated exosomes. Data are presented as means ± SD, *n* = 3. *p < 0.05, **p < 0.01, ***p < 0.001, unpaired t-test.

Given the key role of PD-L2 as an immune checkpoint in immune escape of tumor cells [25], we next explored the effects of TDE-PD-L2 on T cells. We used two different models, Jurkat cells and the isolated lymphocytes from peripheral blood mononuclear cells (PBMC) consisting of principally CD4^+^ and CD8^+^ T cells. Carboxyfluorescein Succinimidyl Ester (CFSE) assays showed that exosomes with PD-L2 KO promoted the proliferation of T cells while exosomes with PD-L2 OE exerted the opposite effects on T cells (Figs. 2A and S3A). Correspondingly, T cells showed increased or decreased activity by exosomes with either PD-L2 KO or OE compared to the control by the assessment of IL-2 and IFN-γ productions (Figs. 2B and S3B), suggesting TDE-PD-L2 negatively regulates the function of T cells in vitro. Furthermore, we also investigated the effects of TDE-PD-L2 on the T cell-mediated cytotoxicity of tumor cells. We established a co-culture system between cancer cells and T cells with feeding exosomes. Exosomes with KO of PD-L2 significantly enhanced cytotoxicity-dependent cancer cell death and decreased the death of T cells in contrast to the control (Figs. 2C and S3C). Conversely, the exosomes with PD-L2 OE had the completely opposite effects on this assay (Figs. 2D and S3D).

Due to the structural similarities and functional redundancies between PD-L1 and PD-L2, as well as their competitive binding to PD-1, we sought to investigate potential differences in the expressions of TDE-PD-L1 and TDE-PD-L2 on immune cells. To this end, we generated PD-L1 (Fig. S4A) and PD-L2 KO (Fig. S4B) strains in two ccRCC cell lines, 786-O and ACHN, respectively. ELISA analysis was conducted to determine the concentrations of PD-L1 and PD-L2 in CTR, PD-L1 KO, and PD-L2 KO cells, as well as in exosomal lysates, with all adjusted to the equal total protein amounts. The ELISA results showed that although cellular PD-L1 levels exceeded those of PD-L2, TDE-PD-L1 levels were significantly lower than TDE-PD-L2 levels (Fig. S4C). Moreover, we assessed the effects of exosomes derived from either PD-L1 KO and PD-L2 KO cells on Jurkat cells. Our findings revealed that PD-L2 KO exosomes had a more pronounced inhibition on Jurkat cells than PD-L1 KO exosomes (Fig. S4D–F), which may be due to high expression of PD-L2 in exosomes. We further investigated the underlying mechanism of TDE-PD-L2-meidated cytotoxicity on cancer cells. We found that CD4^+^ ratio was increased after administration of exosomes with PD-L2 OE in comparison with the control (Fig. 2E). The proportion of CD8^+^ T cells including CTL was also decreased (Fig. 2F). Furthermore, we explored the expression profiles of TDE-PD-L2 on lymphocytes of PBMC, which revealed that TDE-PD-L2 affected the expression of hundreds of genes and was associated with the negative regulation of immune signaling pathways (Fig. S5A–C). Collectively, these data demonstrate that TDE-PD-L2 promotes the death of lymphocytes and decrease the survive of cancer cells in an immune competent condition.

**Fig. 3:**
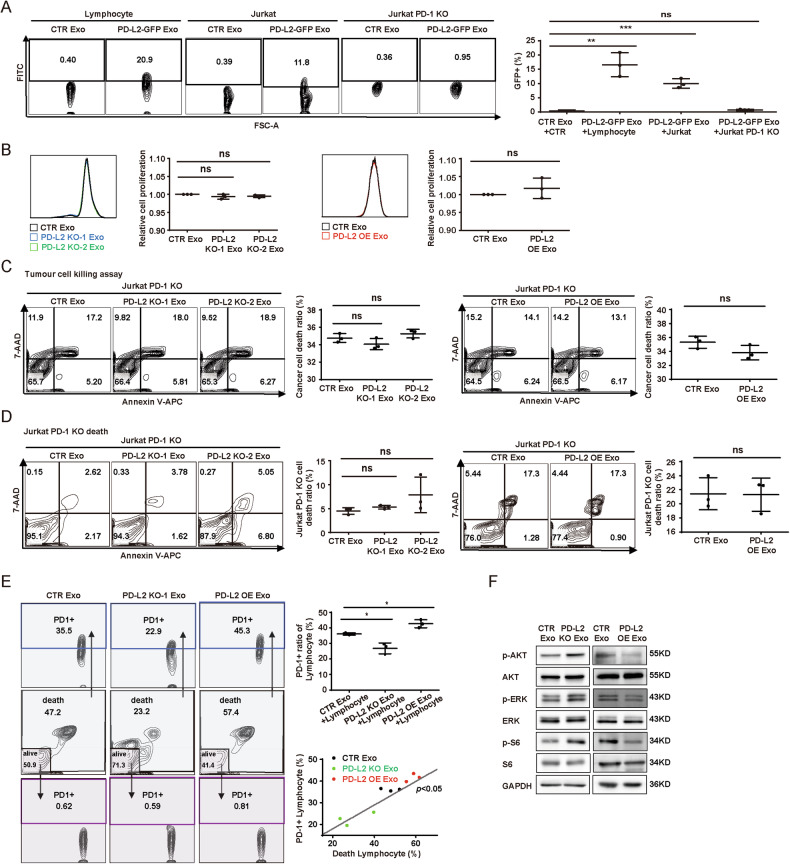
TDE-PD-L2 inhibits the activity and function of Jurkat via binding PD-1. **A** FACS for GFP positive lymphocytes, Jurkat and PD-1 KO Jurkat cells fed with the indicated exosomes for 24 h. **B** Representative FACS and quantification of CFSE proliferation for PD-1 KO Jurkat cells fed with indicated exosomes. **C** Representative FACS and quantification of cancer cell death for PD-1 KO Jurkat cell cytotoxicity assay fed with indicated exosomes. **D** Representative FACS and quantification of death for PD-1 KO Jurkat cell fed with indicated exosomes. **E** Representative FACS and quantification of positive PD-1 ration in lymphocytes incubated with indicated exosomes (right top), and correlation between positive PD-1 and death lymphocytes treaded with the indicated exosomes (right bottom). **F** Immunoblot analysis of the indicated proteins in stimulated Jurkat cells fed with indicated exosomes. Data presented as means ± SD, *n* = 3. *p < 0.05, **p < 0.01, ***p < 0.001, ns, not significant. unpaired t test.

As PD-1 is a receptor of PD-L2 [26], we wondered whether the function of TDE-PD-L2 was dependent on PD-1. FACS was performed on the T cells to examine the binding ability of PD-1 with TDE-PD-L2, which showed that TDE-PD-L2-GFP was significantly increased in both lymphocytes and Jurkat cells but failed to be detected in PD-1 KO Jurkat cells constructed in our previous study [12], suggesting TDE-PD-L2 binds these cells via PD-1 (Fig. 3A). Furthermore, the effects of TDE-PD-L2 on proliferation, apoptosis and cytotoxicity of Jurkat cells were diminished on both PD-1 KO Jurkat cells and PD-1-targeted Nivolumab treatment Jurkat cells (Figs. 3B–D and S6A–D), suggesting TDE-PD-L2 suppresses T cell activation via binding PD-1 similarly to the cell-surface PD-L2. Next, we assessed the effects of TDE-PD-L2 on lymphocytes cells. TDE-PD-L2 levels, surface PD-1 levels of lymphocytes and death ratio of lymphocytes were significantly correlated (Fig. 3E). Altogether, the data suggest that TDE-PD-L2/PD-1 axis plays an antitumor role. Next, we further examined whether TDE-PD-L2 influenced the downstream signaling pathways of PD-1. PD-1 regulates several downstream signaling pathways, including the phosphatidylinositol 3-kinase (PI3K)/AKT, MAPK/ERK1/2, and mammalian target of rapamycin (mTOR) pathways [27]. We wondered whether these pathways are influenced in activated Jurkat cells treated with varying levels of TDE-PD-L2. Immunoblot analysis showed that levels of phospho(p)-AKT, p-ERK1/2 and p-S6 were increased after fed PD-L2 KO exosomes compared to control cells (Fig. 3F).

Given that PD-1/PD-L1 axis plays an important role in tumor-associated macrophages (TAMs) and TAMs regulate immune evasion in ccRCC, we also explored the effects of TDE-PD-L2 on THP-1-derived macrophages, and found that TDE-PD-L2 inhibited the production of the pro-inflammatory cytokine TNF-α (Fig. S7A), reduced the phagocytic capacity of macrophages (Fig. S7B), and promoted their polarization into M2-like suppressive macrophages (Fig. S7C). In summary, TDE-PD-L2 inhibit the activity and function of pro-inflammatory components in lymphocytes and macrophages, contributing to the establishment of an immunosuppressive microenvironment. In conjunction with our previous findings, which indicate that the effects of exosomal PD-L2 on lymphocytes can be rescued by PD-1 antibody treatment and PD-1 KO (Figs. 3B–D and S6A–D), these results suggest that exosomal PD-L2 primarily exerts its effects within the ccRCC immune microenvironment through binding to PD-1.

### TDE-PD-L2 promotes the proliferation and metastasis of ccRCC

**Fig. 4:**
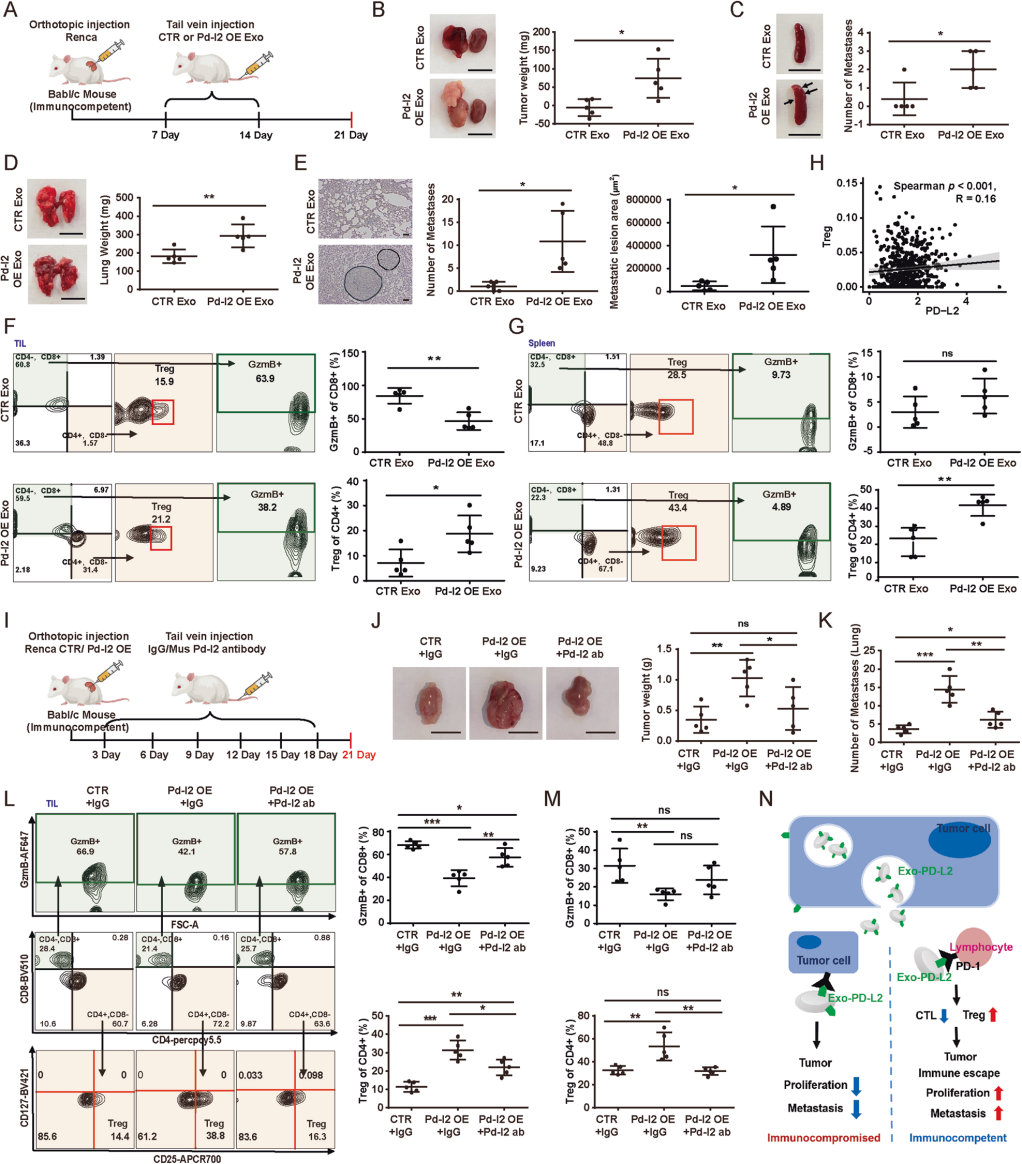
TDE-PD-L2 promotes the proliferation and metastasis of ccRCC in immune competence. **A** Schematic of experimental design for Renca mice cell orthotopic injection into left kidney of normal Babl/c mice, following injection of CTR or PD-L2 OE exosomes into the tail vein and analysis for profiles of immune cells on the indicated time respectively. (*n* = 5). **B** Representative images of tumor foci on the left kidney and the intact right kidney, and the weight of tumor growth evaluated by left kidney weight subtracting right kidney weight. Scale bars, 1 cm. **C** Representative images of brightfield samples (left) and number of metastatic lesions of spleen (right). Arrows, metastatic nodules. Scale bars, 1 cm. **D** Representative images of brightfield (left) and the weight of lungs (right). Scale bars, 1 cm. **E** Representative images of H&E stained samples (left) and number (middle)/area (right) of metastatic lesions. Scale bars, 20 µm. Representative FACS and quantification of CTL (CD8^+^, GzmB^+^) (top) and Treg (CD4^+^, CD127^-^, CD25^+^) (bottom) among CD45^+^, CD3^+^ cells in TIL (**F**) and spleen cells (**G**). **H** Correlation between *PDCD1LG2* mRNA expression and imputed Treg infiltration using KIRC data from TCGA. **I** Schematic of experimental design for CTR or PD-L2 OE Renca mice cell orthotopic injection into left kidney of normal Babl/c mice, following injection of IgG or PD-L2 antibodies into the tail vein and analysis for profiles of immune cells on the indicated time respectively (*n* = 5). **J** Representative images (left) and quantification (right) of tumor foci on the left kidney. Scale bars, 1 cm. **K** Quantification of the number of lung metastatic lesions. Representative FACS and quantification of CTL (top) and Treg (bottom) in TIL (**L**) and spleen cells (**M**). **N** Proposed model for function of TDE-PD-L2 in ccRCC. TDE-PD-L2 promoting the growth and metastasis of ccRCC through systematic increase in Treg and decrease in CTL in immune competence. In contrast, TDE-PD-L2 inhibiting the growth and metastasis of ccRCC without an adaptive immunity. Data presented as means ± SD. *p < 0.05, **p < 0.01, ***p < 0.001, ns, not significant. unpaired t test.

We next explored the potential effects of TDE-PD-L2 on tumor in an immunocompetent mice (Babl/c mice) based on a strategy of murine syngeneic tumor models (Fig. 4A). Exosomes with PD-L2 OE led to a significantly increased weight of tumors in situ and metastasis of lung, as well as more numbers of the metastasis of both lung and spleen (Fig. 4B–E). IHC also revealed that Pd-l2 levels in group of Pd-l2 OE exosomes were significantly increased compared to the control group in kidneys, lungs, and spleens (Fig. S8A), suggesting that TDE-PD-L2 enters these organs to favor tumor growth or metastasis. Furthermore, FACS revealed that TDE-Pd-l2 led to a reduced CTL cell ratio and an increased population of Treg in both tumor-infiltrating lymphocyte (TIL) and spleen (Figs. 4F–G and S8B–C). We further found that the expression levels of *Pdcd1lg2* were associated with the diverse types of TIL in ccRCC from TCGA data, including Treg (Figs. 4H and S8D). Collectively, these data demonstrate that TDE-PD-L2 suppresses immunity and promote the tumor growth and metastasis in immunocompetent mice.

As shown that TDE-PD-L2 mediates the progression of ccRCC tumor (Fig. 4B–E), we hypothesized that antibodies targeting PD-L2 are able to restore effects of TDE-PD-L2 on ccRCC tumor progression. We designed a new strategy using Renca cells with Pd-l2 OE and then injected IgG or Pd-l2-targeted antibodies into the tail vein every 3 days (Fig. 4I). The group with Pd-l2 OE exhibited increased tumor weight, as well as increased number and area of metastatic lesions in lung compared with that of the control group (Figs. 4J, K and S8F). IHC also revealed that Pd-l2 levels were higher in tumor, lung and spleen of Pd-l2 OE group than that of the control group (Fig. S8G), indicating a prerequisite role of TDE-Pd-l2 in distant sites such as the lung and spleen. Analysis of TIL and spleen showed that Pd-l2 OE had led to the increased Treg, and the decreased Cytotoxic T Lymphocyte (CTL) (Fig. 4L, M). These effects of Pd-l2 OE were restored by targeted-Pd-l2 antibodies. Collectively, PD-L2 plays an important role in immune escape of cancer cells via PD-L2 secretion with the increased Treg and decreased CTL.

## Discussion

It is well accepted that PD-L1 is expressed on many types of tumor cells, thus resulting in T cells exhaustion and immune escape of tumor cells [4, 6], so targeting PD-1/PD-L1 axis has achieved significant clinical benefits in multiple cancer types [25, 28, 29]. Many factors have been demonstrated to mediate potential mechanisms of the response or resistance to antibodies targeting PD-1/PD-L1 axis, such as PD-L1 expression levels of tumor cells, TIL, tumor mutation burden, etc. [30, 31]. Clearly, PD-L2 is much less attention in tumor cell escape. Many studies have shown that expression levels of PD-L2 are high in contrast to PD-L1 in multiple cancer types, including RCC [32], cervical cancer [33], and pancreatic ductal adenocarcinoma [34]. It has also revealed that PD-L2 binds to PD-1 with three-fold higher affinity compared to PD-L1 [35]. These studies highlight the potential significance of PD-L2 in immune escape of tumor cells in clinic. However, PD-L2 also binds to repulsive guidance molecule b (RGMb) to regulate respiratory immunity [26] and mediate the resistance of microbiome-related immunotherapy [36]. PD-L2/PD-1 axis controls airway hyperreactivity via regulating number and function of Treg. Collectively, it is highly desirable to investigate the function PD-L2 in tumor progression, develop agents targeting PD-L2 and explore the potential efficiency of these agents in patients with high PD-L2 expression.

It has been well established that exosomes suppress or potentiate tumor progression via various molecules with different underlying mechanisms, one of which is that exosomal PD-L1 promotes tumor progression through direct interaction with PD-1 on the surface of lymphocytes as efficiently as PD-L1 on the tumor cell surface via impairing CTL, enhancing memory immune [37, 38]. Furthermore, TDE-PD-L1 is resistant to targeted-PD-1/L1 immune therapy [31, 37]. However, in this study, we found that TDE-PD-L2 increases tumor metastasis and the systematically increased proportion of Treg via TDE PD-L2. PD-L2 level is lower than PD-L1 level at the cellular level in our tested two lines, while PD-L2 level in exosomes is significantly higher, indicating PD-L2 is preferred to be secreted via exosomes, the underlying mechanism will be able to be explored in future.

Overall, our data show that TDE-PD-L2 suppresses the growth and metastasis of tumor cells in immune compromise and promotes the growth and metastasis of tumor cells in immune-competent (Fig. 4N). This study provides a potential strategy to overcome intrinsic or acquired resistance of PD-1/PD-L1-targted agents.

## Supplementary information

Supplementary information

original western blots

## Data Availability

The authors declare that all data supporting the findings of this study are available within the article and its Supplementary Information files or from the corresponding author upon reasonable request. RNA-seq data have been deposited at GEO and are publicly available as of the date of publication. Accession number is GSE242798.
