# Supplementary material for: PD-L2 of tumor-derived exosomes mediates the immune escape of cancer cells via the impaired T cell function
Source: Cell Death Dis. 2024 Nov 7;15(11):800. doi: 10.1038/s41419-024-07191-7 (PMC11544247; doi:10.1038/s41419-024-07191-7)
Supplement: Supplementary file 1 — Supplementary information [file 41419_2024_7191_MOESM1_ESM.docx]

**Supplementary information for**

**PD-L2 of Tumor-derived Exosomes Mediates the Immune Escape of Cancer Cells via the Impaired T Cell Function**

Tongfeng Liu^1,2^, Shuwen Cheng^2,3^, Bo Peng^2,4^, Haojing Zang^2,5^, Xiaofeng Zhu^1,2^, Xuetong Wang^2,4^, Xujie Zhao^2^, Yinmin Gu^2^, Yongbo Pan^2,6^, Hongbo Hu ^7,^*, Shan Gao^2,^*

^1^Medical College, Guizhou University, Guiyang 550025, China

^2^Zhongda Hospital, School of Life Sciences and Technology, Advanced Institute for Life and Health, Southeast University, Nanjing 210096, China

^3^Medical School of Nanjing University, Nanjing 210046, China

^4^School of Biomedical Engineering (Suzhou), Division of Life Sciences and Medicine, University of Science and Technology of China, Hefei 230000, China

^5^Department of Microbiology and Immunology, Shanxi Medical University, Taiyuan, Shanxi, 030001, China

^6^Guangdong Cardiovascular Institute, Guangdong Provincial People's Hospital, Guangdong Academy of Medical Sciences

^7^Center for Immunology and Hematology, Department of Biotherapy and Cancer Center, State Key Laboratory of Biotherapy, West China Hospital, Sichuan University, Chengdu 610041, China

*Corresponding author: Shan Gao. E-mail: gaos@sibet.ac.cn. Hongbo Hu. E-mail: hongbohu@scu.edu.cn

**CONTENTS**

**Supplementary Materials and Methods**

Method details

**Supplementary Figures**

Fig. S1: PD-L2 is Expressed on ccRCC.

Fig. S2: Cellular PD-L2 and TDE-PD-L2 Inhibited the Proliferation and Metastasis of ccRCC in Immunodeficient.

Fig. S3: TDE-PD-L2 Inhibits the Activity and Function of Jurkat.

Fig. S4: Comparison of Inhibitory Effects of ccRCC TDE-PD-L2 and TDE-PD-L1 on Jurkat Cells.

Fig. S5: TDE-PD-L2 Inhibits the Activity and Function of Lymphocytes.

Fig. S6: TDE-PD-L2 Inhibits the Activity and Function of Jurkat via binding PD-1.

Fig. S7: TDE-PD-L2 Inhibits the Activity and Function of THP-1-derived macrophages.

Fig. S8: TDE-PD-L2 systematic increase in Treg and decrease in CTL.

**Supplementary Tables**

Table S1: Reagent or source

Table S2: Primers and target sequences

**Supplementary Materials and Methods**

**Method details**

***RNA-sequence and analysis***

Exosomes were isolated from equal numbers of 786-O CTR and PD-L2 KO cells (1×10^8^) culture supernatants. These exosomes were then administered to PMA (2mM) and Ionomycin (20 ng/ml) stimulated peripheral blood lymphocytes (5×10^5^) in 10cm Petri dishes for a duration of 72 hours. RNA-sequence analysis was performed using Illumina NovaSeq 6000. Raw read counts were utilized for differential gene expression analysis through DESeq2. Gene expression levels were measured by the number of uniquely mapped fragments per kilobase of transcript per million mapped reads (FPKM). Differential expression genes (DEGs) between lymphocytes treated with CTR or PD-L2 KO exosomes were determined using the following criteria: p < 0.05 and |log2FC| > 1. Gene Set Enrichment Analysis (GSEA) was conducted to analyze the gene expression profiles in Gene Ontology (GO) and Kyoto Encyclopedia of Genes and Genomes (KEGG) database. The RNA sequence data from this study have been deposited in the GEO database under accession number GSE242798.

***Immunohistochemistry***

Lung, spleen and tumor tissue was fixed in 10% formalin overnight. The fixed tissues were processed into paraffin blocks, cut into 5μm sections, and mounted onto slides. The slides were then placed in a drying oven at 37ºC for 8 hours to dry. Sections were dewaxed with Xylene and hydrated in 100%, 90%, 70% and 50% ethanol sequentially. The slides were incubated with 3% hydrogen peroxide for 10 min, followed by antigen retrieving using 0.01 M citrate buffer (pH 6.0) for 30 min. After blocking with 5% BSA, slides were incubated overnight at 4 °C with relevant primary antibody. Detailed information about the antibodies can be found in TableS1. After that, the secondary biotin-conjugated antibody was applied for 2 hours at room temperature. 1×DAB substrate was applied to the sections after two 5 minutes PBS-T washes. The procedure was completed to yield a brown colored product. Slides were counterstained with hematoxylin, dehydrated, and permanently mounted.

***Immunofluorescence***

The cells were grown on 35mm Nunc™ Glass Bottom Dishes for 24 hours. After this incubation period, the cells were fixed with 4.0% paraformaldehyde and permeabilized with 0.5% Triton X-100 (Sigma, Cat#9036-19-5) at room temperature. The cells were blocked in 5% BSA for 1 hour. Then, immunofluorescence was performed using an anti-CD63 and PD-L2 antibody for 12 hours at 4°C. To label the primary antibodies, the cells were washed with cold PBS and then incubated with Alexa Fluor 488-conjugated goat anti-rabbit IgG and Alexa Fluor 594-conjugated goat anti-mouse IgG for 2 hours at room temperature. To visualize the nuclei, the cells were counter-stained with 4’-6-diamidino-2-phenylindole (DAPI). Finally, the cells were observed using a fluorescence microscope at a magnification of 60×. Three independent experiments were performed, and three random sights were chosen in each sample for analysis. Colocalization coefficient were analyzed by ImageJ plugin Coloc 2. The antibodies were listed in TableS1.

***Plasmid transfection and siRNA knockdown***

For lentivirus-mediated RNA interference, the lentivirus vectors containing the shRNAs were transfected into 786-O cells. Lentivirus was produced by co-transfecting the specific plasmids with packaging vectors PMD2.G and psPAX2 in a ratio of 8:3:6 into HEK293T cells. The supernatant was collected 72 and 96 hours after transfection for infection. Stable 786-O cell lines were selected using puromycin (1mg/ml) or geneticin (G418, 120 µg/ml).

***Construction of Knockout Cell Lines***

Electroporation was optimized to achieve an efficiency greater than 30%. The knockout vector was designed using CRISPR Design Tool and constructed using the pGK1.1 vector as the backbone. The vector was transfected into the cells via electroporation. High-efficiency knockout cell pools were selected and single clones were grown in a 96-well plate. Genomic DNA was extracted using the Genloci DNA extraction kit (Invitrogen, Cat#K182002). PCR was used to sequence the deletion site adjacent sequences, and the gene knockout detection kit (Cruiser, Cat#GP0102) was used to screen for positive clones. Positive clones were validated through sequencing, and clones from two different parental lines exhibiting different knockout conditions were further confirmed through Thymine Adenine cloning.

***Purification of exosomes***

Exosomes purification from cell culture supernatants involved the following steps: Firstly, cells were cultured in media supplemented with 10% exosome-depleted FBS (Gibco, Cat#A27208-03) for 48 hours. Next, the culture supernatants were centrifuged at 2,000g for 20 minutes to remove cell debris and dead cells. The resulting supernatants were collected and subjected to a further centrifugation step (Eppendorf, Centrifuge 5810 R) at 15,000 g for 30 minutes, aimed at eliminating large extracellular vesicles. Subsequently, the supernatants were filtered through a 0.22μm filter membrane (Merck, Cat# SLGP033NS), and the filtrates were concentrated to 5ml using a 100kDa ultrafiltration tube (Merck, Cat#UFC910024). Following this, the concentrated supernatants were centrifuged (Beckman Coulter Avanti J30I) at 110,000 g for 2 hours. All centrifugation steps performed at 4 °C. The resulting exosomes were then sedimented, re-suspended, and washed in PBS. This was followed by another round of ultracentrifugation, and the resulting pellets were finally resuspended in 100 μL PBS.

***Isolation of*** ***free PD-L2 protein and PD-L2 of extracellular vesicles***

Free PD-L2 protein and nano-sized extracellular vesicle PD-L2 were purified from cell culture supernatants. Cells and debris were removed by centrifugation at 2,800 g for 20 minutes. The resulting supernatants were then subjected to further centrifugation at 10,000 g for 30 minutes to precipitate the larger extracellular vesicles. The remaining supernatants were used to isolate the free PD-L2 protein through a two-step ultrafiltration process using ultrafiltration centrifugal tubes with molecular weight cutoffs of 100 kDa and 30 kDa.

Separation of MV and exosomes was conducted as previously reported (1). To separate MV and exosomes, iodixanol density gradient centrifugation was employed. Solutions of different densities (60%, 50%, 40%, 30%, 25%, 15%, 10%, and 5%) were created by diluting a stock solution of OptiPrep™ (60% aqueous iodixanol from Sigma) in a mixture of 0.25 M Sucrose, 0.9 M NaCl, and 120 mM HEPES at pH 7.4. The filter residue from the 100 kDa ultrafiltration centrifugal tube was mixed with the bottom layer of the iodixanol density gradient, and subsequent solutions were carefully layered on top. Centrifugation was performed at 100,000 x g for 4 hours at 4 °C using a Beckman Coulter Avanti J30I centrifuge. The top four individual fractions and the remaining fractions were collected separately, washed with PBS, and pelleted by centrifugation at 100,000 x g for 1 hour at 4 °C. The resulting pellets from each fraction were then suspended in PBS.

The isolated MV and exosomes were analyzed using nanoparticle tracking analysis (NTA) to determine their number and size distribution. The proportion of PD-L2 in each fraction of the extracellular matrix of two high invasion metastasis ccRCC cell lines were quantified using an enzyme-linked immunosorbent assay (ELISA).

***Characterization of purified exosomes***

For verification of purified exosomes using transmission electron microscopy (TEM), we applied purified exosomes suspended in PBS onto formvar carbon-coated nickel grids. The grids were then stained with 2% uranyl acetate, air-dried, and examined using a JEM-1011 transmission electron microscope. After each staining step, we performed five washes with PBS and five washes with ddH_2_O before contrast staining with 2% uranyl acetate.

To determine the size and concentration of exosomes purified from cell culture supernatants or patients’ tumor interstitial fluid, we used NTA with a ZetaView Twin system (Particle metrix). This system is equipped with fast video capture, particle-tracking software, and two lasers (405nm and 488nm) for accurate measurements.

***Cancer cell proliferation, migration assay***

For the cell proliferation assay, the cells were seeded in 96-well plates at a density of 1000 cells per well in 100 μl of cell suspension. The cell viability was measured using the CellTiter-Glo Luminescent Cell Viability Assay (Promega, Cat# G7570) after 0, 3, and 5 days in culture.

For the exosome treated cancer cell migration assay, 1×10^5^ 786-O cells were seeded in 6 wells of a low adhesion dish (Corning, Cat#3471) and fed with PD-L2 CTR/KO/OE exosomes at a concentration of 1×10^10^ exosomes. The cells were incubated for 24 hours, washed, and then counted. The subsequent procedures were carried out following the same steps as the cell migration assay.

For the cell migration assay, 2×10^4^ cells in serum-free medium were added to the upper chamber of an 8 μm 24 well Transwell (Corning, Cat#3412). Then, 600 μl of medium supplemented with 10% FBS were added to the lower chamber as a chemotactic agent. The cells were cultured at 37 °C in a humidified incubator with 5% CO^2^ for 24 hours. After incubation, the cells that migrated through the filters were fixed with methanol and stained with crystal violet. Three non-overlapping fields of view were selected under a microscope to calculate the total number of migrated cells.

***ELISA***

The levels of IL-2 and IFN-γ were measured using ELISA in lymphocyte and Jurkat cell culture supernatants after 72 hours of feeding with 786-O PD-L2 CTR/KO/OE (1×10^10^) exosomes in a 6-well co-culture plate (Corning, Cat#3422). The levels of TDE-PD-L2 were also estimated using ELISA from the cell culture supernatants and tumor interstitial fluid. The levels of cellular PD-L2 were estimated using ELISA, with equal concentrations and volumes of protein lysis fluid. The ELISAs were performed following the manufacturers’ instructions. The ELISA kits used were listed in TableS1.

To determine the amounts of exosomal PD-L1 or PD-L2, the standard curve for either PD-L1 or PD-L2 was respectively constructed based on ELISA kits for PD-L1 and PD-L2 from Thermo Fisher Scientific (BMS2212 and BMS2215). Then the protocol of these kits was used to determine their amounts based on the above standard curve.

***CFSE assay***

To assess for the proliferation of lymphocytes and Jurkat cells, CFSE, a dye for the tracking of cell division (Thermo Fisher Scientific, Cat#C34554) was used. A total of 1 × 10^6^ lymphocytes or Jurkat cells were stained with CFSE at 5 μM. The cells were then incubated at 37°C for 20 minutes and the reaction was stopped by adding 5 volumes of cold medium with 10% FBS. Then, 1 × 10^5^ CFSE-labeled lymphocytes or Jurkat cells were seeded in a 6-well plate and fed with 1 × 10^10^ exosomes with different PD-L2 expressions for 96 hours. The intensity of CFSE staining was scored based on the median fluorescence intensity (MFI) of FITC measured and analyzed by flow cytometry. Quantification of lymphocyte and Jurkat cells proliferation as 1/ MFI.

***T cell-mediated tumor cell killing assay***

To assess the impact of ccRCC tumor cell-derived exosomes (TDE) on the cytotoxic function of lymphocytes and Jurkat cells against tumor cells, the isolated lymphocyte cells were obtained from PBMC and plated at a concentration of 5×10^5^ cells/ml in a 10 cm plate. The lymphocytes were then stimulated with PMA (2 mM) and Ionomycin (20 ng/ml) for 6 hours, until the proportion of CD69^+^ cells reached over 75%. The stimulated lymphocytes or Jurkat (4 × 10^4^ cells/well) were treated with CTR, PD-L2 OE or KO exosomes (1×10^10^/well), and co-cultured with CFSE-labeled 786-O cells (4×10^4^) in 6-well plates, maintaining an effector to target (E: T) ratio of 1:1. After 72 hours of co-culture, both floating and adherent cells were collected, stained with Annexin V-APC and 7-AAD (Biolegend, Cat# Cat#640930), and analyzed using flow cytometry. The primary antibodies used are listed in TableS1. The ratio of cell death for 786-O or lymphocytes was calculated using the formula: (1- Annexin V^-^,7-AAD^-^) ×100%

***The exosomes /T cell binding assay***

To investigate the physical interactions between TDEs and lymphocyte cells, we treated stimulated lymphocytes, WT, or PD-1 KO Jurkat cells (2 × 10^5^ cells/well) with purified exosomes (1×10^10^) obtained from 786-O PD-L2-GFP OE cells or empty vector control cell culture supernatants for 4 hours in a 6-well plate. Next, the cells were fixed in 4% paraformaldehyde at room temperature for 15 minutes, washed twice with 10 ml of PBS, and pelleted by ultracentrifugation (2000rpm,5 minutes). The proportion of exosomes bound to lymphocytes was determined by measuring the positive rate of FITC using flow cytometry.

***Preparation of macrophages derived from THP-1 cells***

Centrifuge the THP-1 cells in the logarithmic growth phase and resuspend them in RPMI-1640 culture medium. Add PMA and count the cells under a microscope, adjusting the THP-1 cell density to 5 × 10^5^ cells/mL. Set the final concentration of PMA to 100 ng/mL and seed the cells into a six-well plate, adding 2 mL of cell suspension to each well. After 48 hours, observe the cells under a microscope to confirm the successful differentiation into macrophages. Following PMA induction, the monocyte THP-1 transitions from suspension growth to adherent growth, changing from a rounded to an irregular shape, with further increases in cell volume. The cytoplasm becomes less dense, and the nucleus enlarges significantly, with numerous distinct organelles becoming visible. A small number of projections can be observed around the cell membrane.

***Assessment of the phagocytic ability of macrophages***

After incubating macrophages labeled with CFSE with different exosomes for 24 hours, CM-Dil was used to label Jurkat cells. The two cell types were co-cultured for an additional 24 hours, followed by repeated washing to remove any unphagocytosed suspended Jurkat cells. Co-localization was assessed using a fluorescence microscope, while cells were digested with 0.25% EDTA and subsequently analyzed for the proportion of red fluorescent macrophages via flow cytometry. Evaluate macrophage phagocytic ability by the proportion of internally labeled Jurkat cells; a higher Red+% reflects enhanced phagocytic capacity.

***Animal study***

In syngeneic model experiments, 6-8-week-old male NOD SCID Balb/c mice or normal Balb/c mice were used. The mice were anesthetized by injecting Pentobarbital Sodium (50mg/kg) into the abdominal cavity. The chest and abdomen hair of the mice were shaved using a razor. A surgical incision of less than 1 cm was made on the left side of the back to expose the left kidney. Renca cells were harvested in PBS (1×10^7^cells/ml), and a 100 μl cell suspension was injected into the left kidney of the SCID mouse following standard injection procedures. After the injection, pressure was applied for 10 seconds upon needle extraction to prevent leakage, and then the wounds were sewn.

After successful establishment of the in-situ renal cancer model, mice were subjected to exosomes treatment via injection into the tail vein at day 7 and 14 following tumor injection. To obtain exosomes, 1.5 × 10^8^ CTR and Mus-Pd-l2 OE Renca cells were seeded in ten 15 cm dishes (Corning, Cat#430599) and cultured for 48 hours. Exosomes were isolated as described earlier. CTR and Mus-Pd-l2 OE exosomes were resuspended in 500 μl of PBS. Each mouse was injected with 100 μl of PBS containing the respective exosomes.

To carry out the antibody treatment experiment, briefly, 1×10^6^ CTR or Mus-Pd-l2 OE Renca cells were orthotopically injected into the left kidney of normal Balb/c mice. Following that, 100 µg of IgG or Mus-Pd-l2 (BioXCell, Cat#BP0089, Cat#BE0112) antibodies, dissolved in 100 µl of PBS, was administered via tail vein injections on day 3, 6, 9, 12, 15, and 18 after the operation, respectively.

ccRCC tumor growth was assessed by measuring the tumor weight, calculated as the difference between the weight of the left kidney and the weight of the right kidney. The degree of lung metastasis was evaluated by counting the number of metastatic lesions. The number of metastatic lesions in mice were determined by examining H&E staining sections of each sample under a microscope at 40× magnification, with 3 random fields analyzed for each section.

All protocols involving animals were previously approved by the Ethics Committee for the Use of Experimental Animals of Suzhou Institute of Biomedical Engineering and Technology, Chinese Academy of Sciences (Suzhou, Jiangsu, China).

***Isolation and analysis of TILs and spleen cells***

Tumor tissues from Renca tumor-bearing mice were digested using a mechanical method to obtain a single cell suspension. The tumor tissues were manually dissected into 1-3 mm pieces using a surgical scalpel. The tissues were then chopped into single cells by gently grinding through 70-μm filters and 200 μm nylon mesh, respectively. The Mouse TIL Isolation Kit (Solarbio, Cat#p9000) was utilized to remove cancer cells and enrich leukocytes from the tumor tissue. The isolation procedure was carried out according to the instructions provided by the manufacturer.

Spleens were surgically removed using sterilized surgical equipment. The spleens were weighed and crushed using the blunt end of a 1 mL syringe on Petri dishes containing 5 mL of PBS. The spleen mixtures were then individually filtered through a 70 μm filter into a 50 mL conical tube. The tubes were centrifuged at 2000 rpm for 5 minutes at room temperature. After the centrifugation, the cell pellets were resuspended in 5 mL of red blood cell lysis solution (Solarbio, Cat#R1010-500ml) on ice for 5 minutes. The lysis was stopped by adding 30 mL of PBS. After another wash, the cells were filtered through a 200 mesh filter into 15 mL conical tubes. Finally, the cells were washed again and counted.

Flow cytometry staining was performed on the processed cells according to the specific steps outlined in the method Flow cytometry. After staining, lymphocytes were gated based on side scatter (SSC-A) and forward scatter (FSC-A) to exclude debris and non-lymphocytic cells. The cells were then further gated using forward scatter (FSC-A) and side scatter (FSC-H) to exclude doublets. Live cells were gated based on PE-CF594^-^. CD4^+^ and CD8^+^ tumor-infiltrating lymphocytes (TILs) were further gated on CD45^+^CD3^+^ cells. The following monoclonal antibodies specific to the indicated antigens were used for the gating: BV786-CD45, BV650-anti-CD3, PerCP-Cy™5.5-anti-CD4, BV510-anti-CD8, APC-R700-anti-CD25, BV421-anti-CD127, and Alexa Fluor® 647 anti-Granzyme B. Detailed information about the antibodies can be found in TableS1. Flow cytometry data analysis was performed using FlowJo software.

***Flow cytometry***

General flow cytometry staining protocol was as follows. All samples were first blocked in FACS Buffer (PBS+2%BSA) containing FcBlock (1µg/ml) for 10 min at room temperature. Live cells were identified and gated using Fixable Viability Stain 620 (BD, Cat#565388) staining on ice for 30 minutes. Antibodies were added at indicated concentrations and samples were incubated at room temperature for 45 minutes. Samples were then washed with FACS Buffer and acquired on a BD FACSCelesta immediately.

For intracellular staining, samples were stained for surface antigens and viability, then cells were fixed and permeabilized according to manufacturer’s instructions (BD, Cat#554714). Cells were resuspended in FACS buffer prior to acquisition.

**Supplementary Figures**


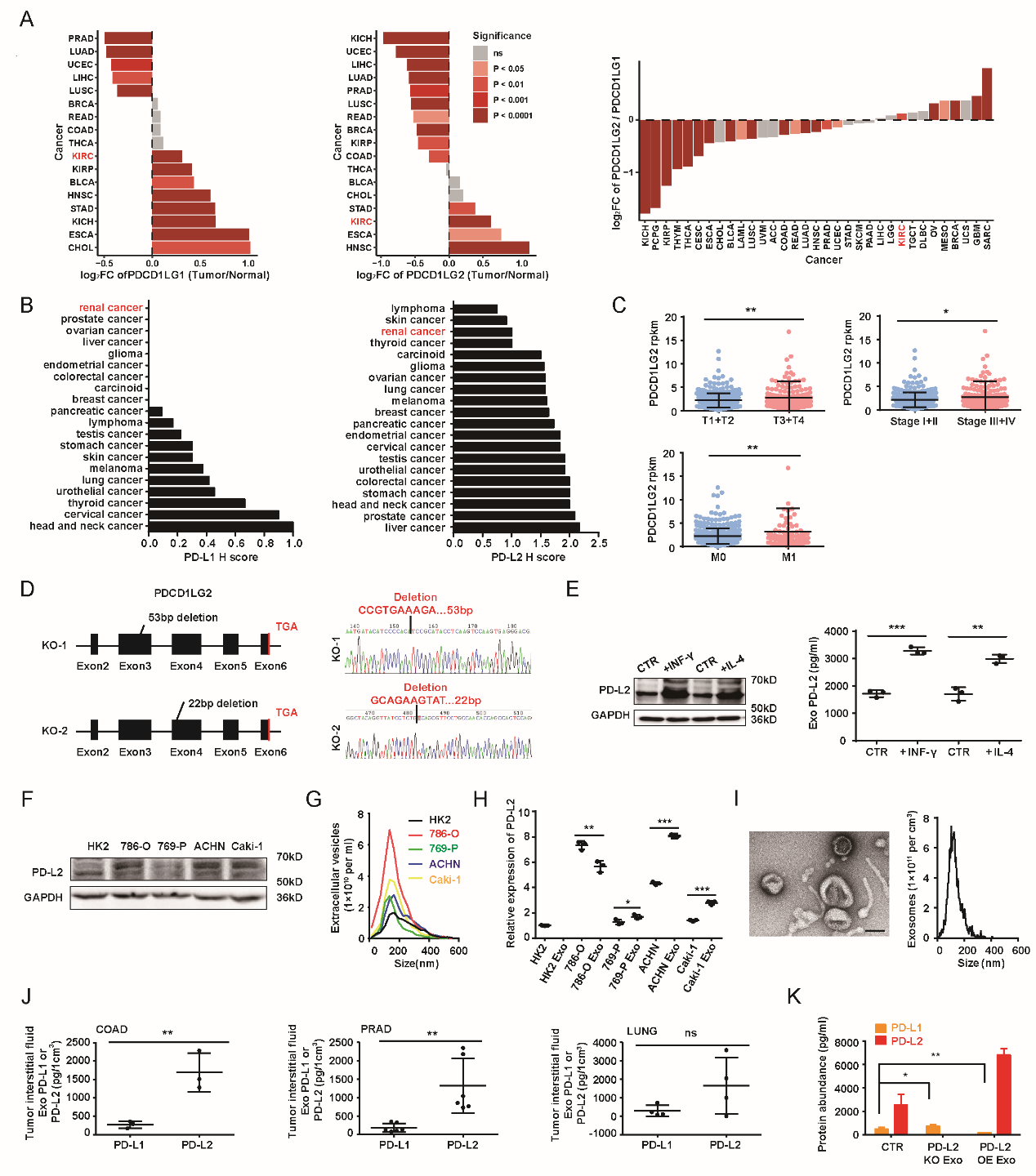


**Fig. S1 | PD-L2 is Expressed on ccRCC. Related to Figure 1.** (A) *PDCD1LG1* (left) and *PDCD1LG2* (middle) mRNA expression in cancer tissues relative to normal tissues and relative expression levels of PD-L2/PD-L1 (right) in TCGA data. (B) PD-L1 (left) and PD-L2 (middle) protein expression in HPA pathology data. H score represents the score of either PD-L1 or PD-L2 immunochemistry staining from the HPA pathology data. (C) Comparison mRNA expression of *PDCD1LG2* expression between ccRCC tissues and adjacent normal tissues base on TCGA data. The expression of PD-L2 is grouped by T stage (top left), tumor differentiation (top right) and M stage (bottom left). (D) Strategy used to create the PD-L2 KO 786-O cell line. Alignment of mutant sequence containing a 53bp or a 22bp deletion in PD-L2 exon 3 and exon 4 using the Cas9 (left). Sequencing peak diagram of Human-PD-L2 KO 786-O showing the missing part of PD-L2 DNA (right). (E) Immunoblot and ELISA analysis for cellular PD-L2 (left) and TDE-PD-L2 (right) in 786-O treated with indicated cytokines. (F) Immunoblot analysis for the cellular PD-L2 protein expression in the immortalized human HK2 tubular epithelial cell line (normal control) and the indicated ccRCC cell lines. (G) Representative NTA images of extracellular vesicles purified from HK2 and ccRCC cell lines culture supernatants. (H) ELISA analysis for PD-L2 in whole cell lysate and exosomal lysate from different ccRCC cell lines (n=3). (I) The characterization of exosomes isolated from tumor interstitial fluid using TEM (left) and NTA (right). Scale bars, 100nm. (J) ELISA analysis for exosomes PD-L2 from tumor interstitial fluid (right) from Colon Cancer (COAD, n=3), Prostate Cancer (PRAD, n=6) and Lung Adenocarcinoma (LUAD, n=4) patients. (K) ELISA analysis for PD-L1 in exosomes isolated from 786-O cells with differential PD-L2 expression levels (n=3). Data are presented as means ± SD. *p<0.05, **p<0.01, ***p<0.001. ns, not significant. unpaired t test.


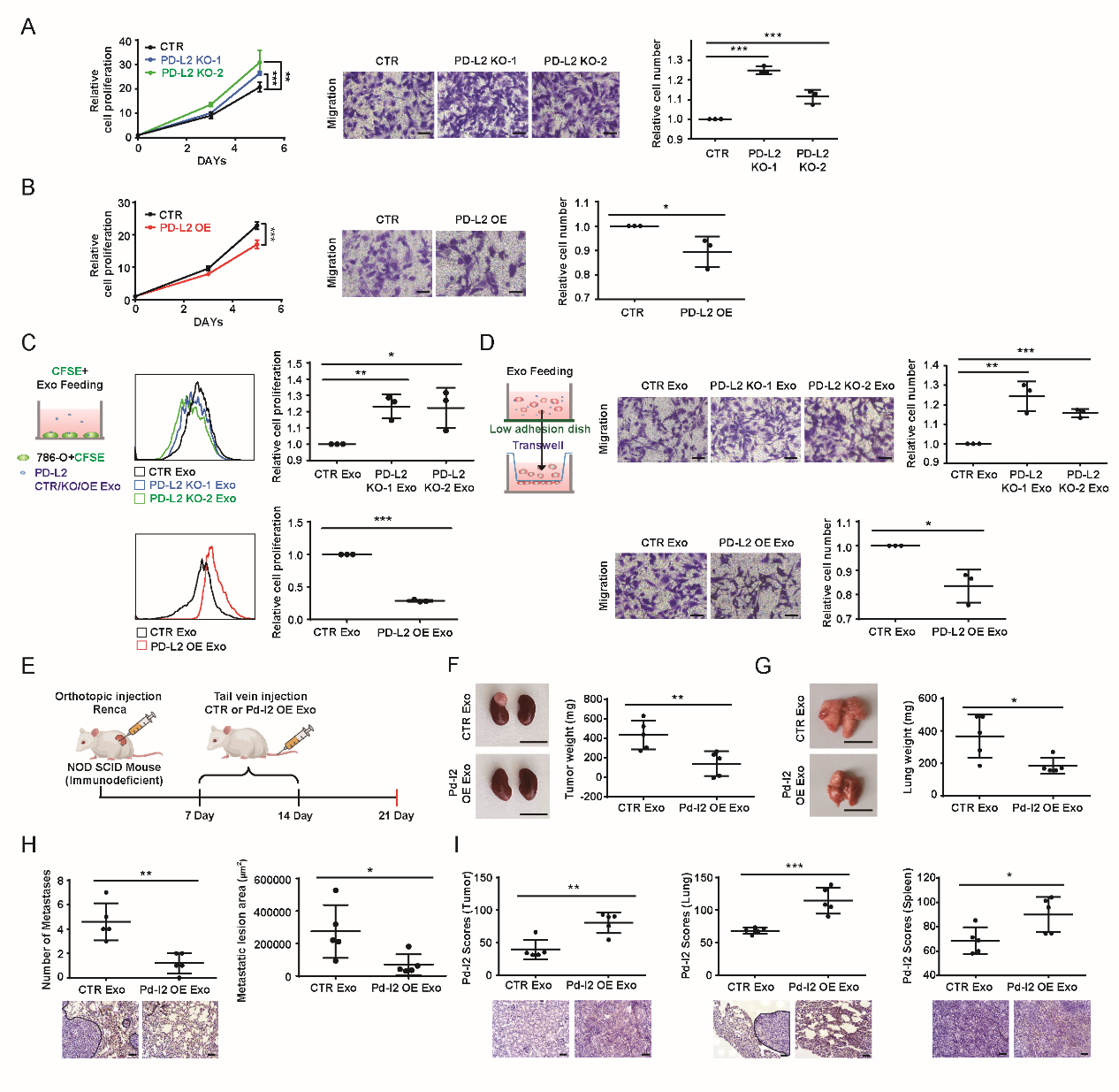


**Fig. S2 | Cellular PD-L2 and TDE-PD-L2 Inhibited the Proliferation and Metastasis of ccRCC** **in Immunodeficient**. (A-B) The relative proliferation (left), representative micrographs (middle) and the quantitated relative cell number (right) of transwell assays assessing CTR and PD-L2 KO (A) or PD-L2 OE (B) 786-O cells. Scale bars, 20 µm. Cell proliferation assay was calculated using two-way ANOVA. (C) Schematic of experimental strategy (left), representative FACS (middle), and quantification (right) of 786-O proliferation as 1/ mean fluoresce intensity (MFI) treated with the indicated exosomes. (D) Schematic of experimental strategy (left)，representative transwell micrographs (middle) and the quantitated relative cell number of 786-O treated with the indicated exosomes. Scale bars, 20 µm. (E) Schematic of experimental design for Renca mice cell orthotopic injection into left kidney of NOD SCID Babl/c mice, and following injection of CTR or PD-L2 OE exosomes into the tail vein on day 7 and 14 days respectively. (n=5). (F) Representative images of tumor foci on the left kidney and the intact right kidney, and the weight of tumor growth evaluated by left kidney weight subtracting right kidney weight. Scale bars, 1cm. (G) Representative images of brightfield (left) and the weight of lungs (right). Scale bars, 1cm. (H) Representative images of H&E-stained samples (bottom left) with scale bars of 20 µm. Additionally, the quantification of the number (top left) and area (top right) of metastatic lesions is shown. (I) Representative images (top) and quantification (bottom) of PD-L2 in ccRCC in left kidney (left), lung (middle), spleen (right) as determined by IHC analysis. Scale bars, 20 µm. Data presented as means ± SD. *p<0.05, **p<0.01, ***p<0.001.


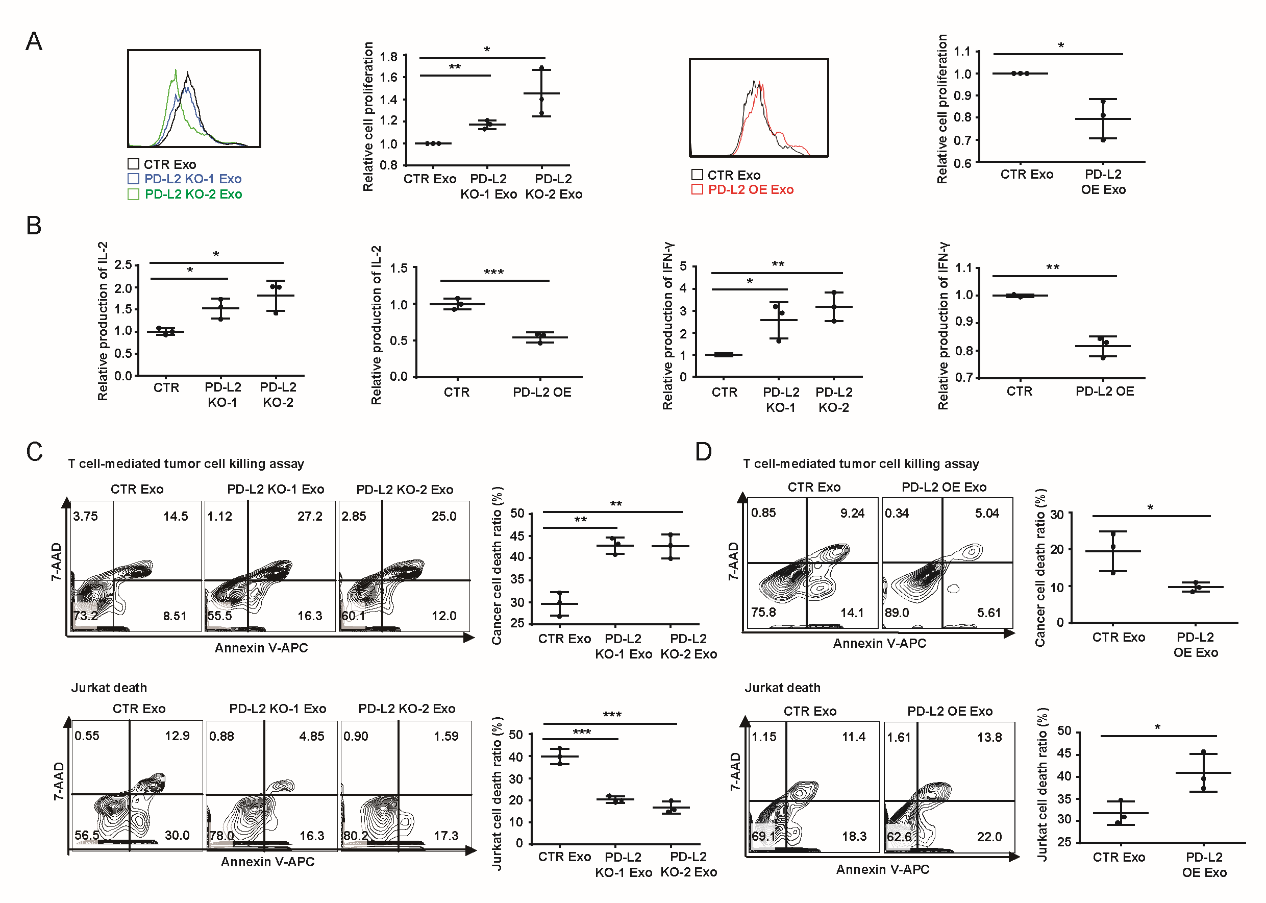
 **Fig. S3 |** **TDE-PD-L2 Inhibits the Activity and Function of Jurkat.** **Related to Figure 2.** (A) Representative FACS and quantification of Jurkat cell proliferation as 1/MFI treated with the indicated exosomes. (B) Quantification of IL-2 (left) and IFN-γ (right) levels of the Jurkat cell treated with the indicated exosomes. (C-D) Representative FACS and quantification of death for cancer cells (top) and Jurkat cells (bottom) fed with the the CTR and PD-L2 KO (C) or PD-L2 OE (D) exosomes. Data presented as means ± SD, n=3. *p<0.05, **p<0.01, ***p<0.001.


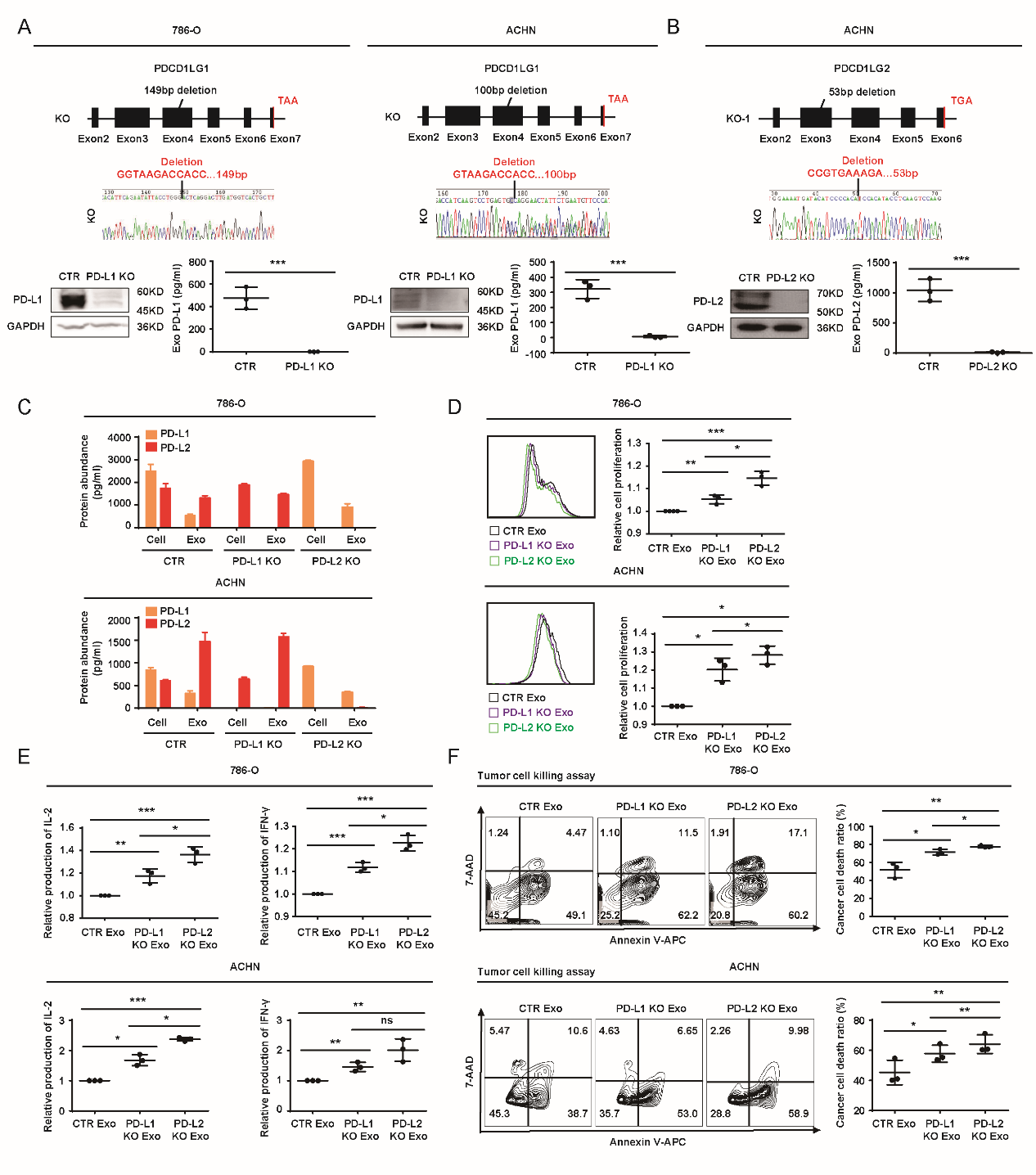


**Fig. S4 | Comparison of Inhibitory Effects of ccRCC TDE-PD-L2 and TDE-PD-L1 on Jurkat Cells. Related to Figure 2.** (A) Strategy used to create the PD-L1 KO 786-O (left)/ACHN (right) cell line (top). Sequencing peak diagram of Human-PD-L1 KO 786-O (left)/ACHN (right) showing the missing part of PD-L1 DNA (middle). Immunoblot (left bottom) and ELISA analysis (right bottom) for the KO efficiency of PD-L1 in 786-O (left)/ACHN (right) cells. (B) Strategy used to create the PD-L2 KO ACHN cell line (top). Sequencing peak diagram of Human-PD-L2 KO ACHN showing the missing part of PD-L2 DNA (middle). Immunoblot (left bottom) and ELISA analysis (right bottom) for the KO efficiency of PD-L2 in ACHN cells. (C) ELISA analysis of PD-L1 (orange) and PD-L2 (red) in the cellular and exosomal lysates of CTR, PD-L1 KO, and PD-L2 KO 786-O (top) and ACHN (bottom) cells, all adjusted to equal total protein amounts. (D) Representative FACS and quantification of Jurkat cell proliferation as 1/MFI treated with the indicated exosomes isolated from 786-O (top)/ ACHN (bottom). (E) Quantification of IL-2 (left) and IFN-γ (right) levels of the Jurkat cell treated with the indicated exosomes isolated from 786-O (top)/ ACHN (bottom). (F) Representative FACS for death ratio of cancer cells fed with the indicated exosomes isolated from 786-O (top)/ ACHN (bottom). Data are presented as means ± SD, n=3. *p<0.05, **p<0.01, ***p<0.001, unpaired t-test.


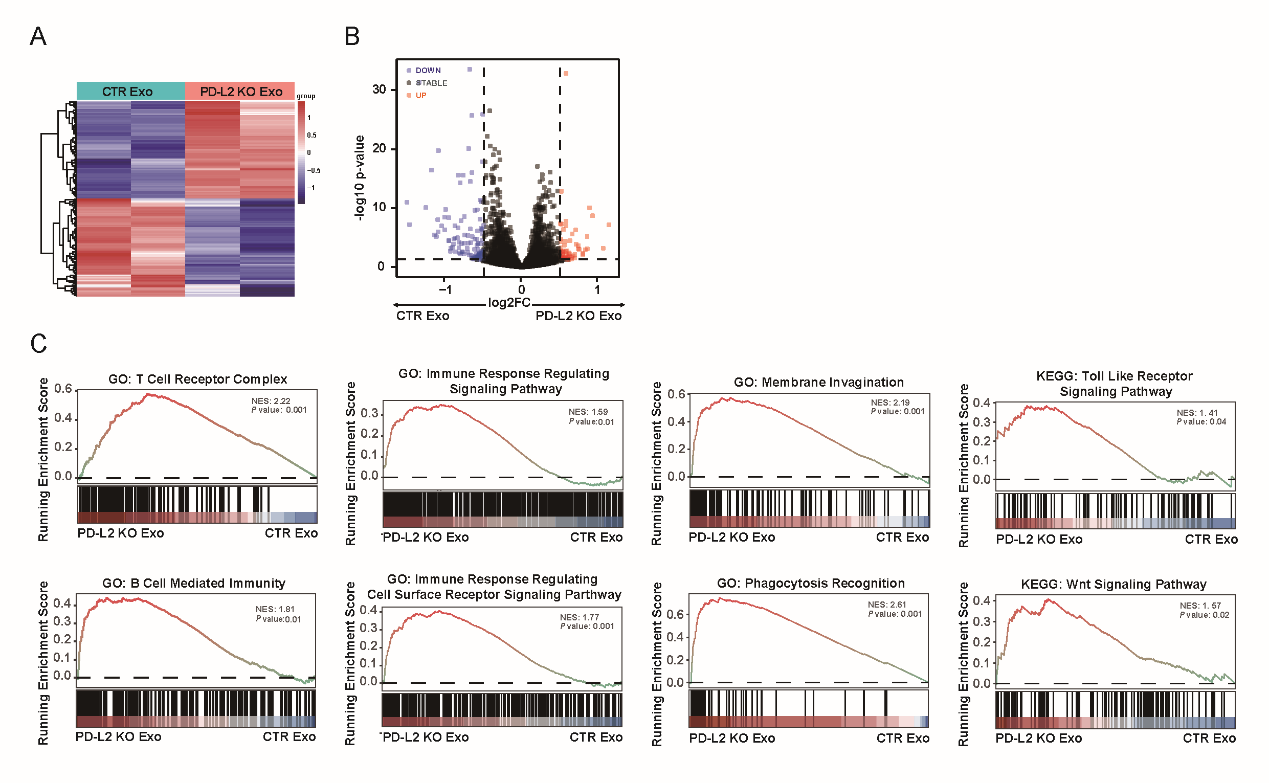
 **Fig. S5 | TDE-PD-L2 Inhibits the Activity and Function of Lymphocytes. Related to Figure 2.** (A-B) Heat map (A) and volcano plot (B) of RNA-seq showing the DEGs of lymphocytes fed with indicated TDE-PD-L2. (C) GSEA analysis showing the enrichment of “regulation of immune signaling pathways”.


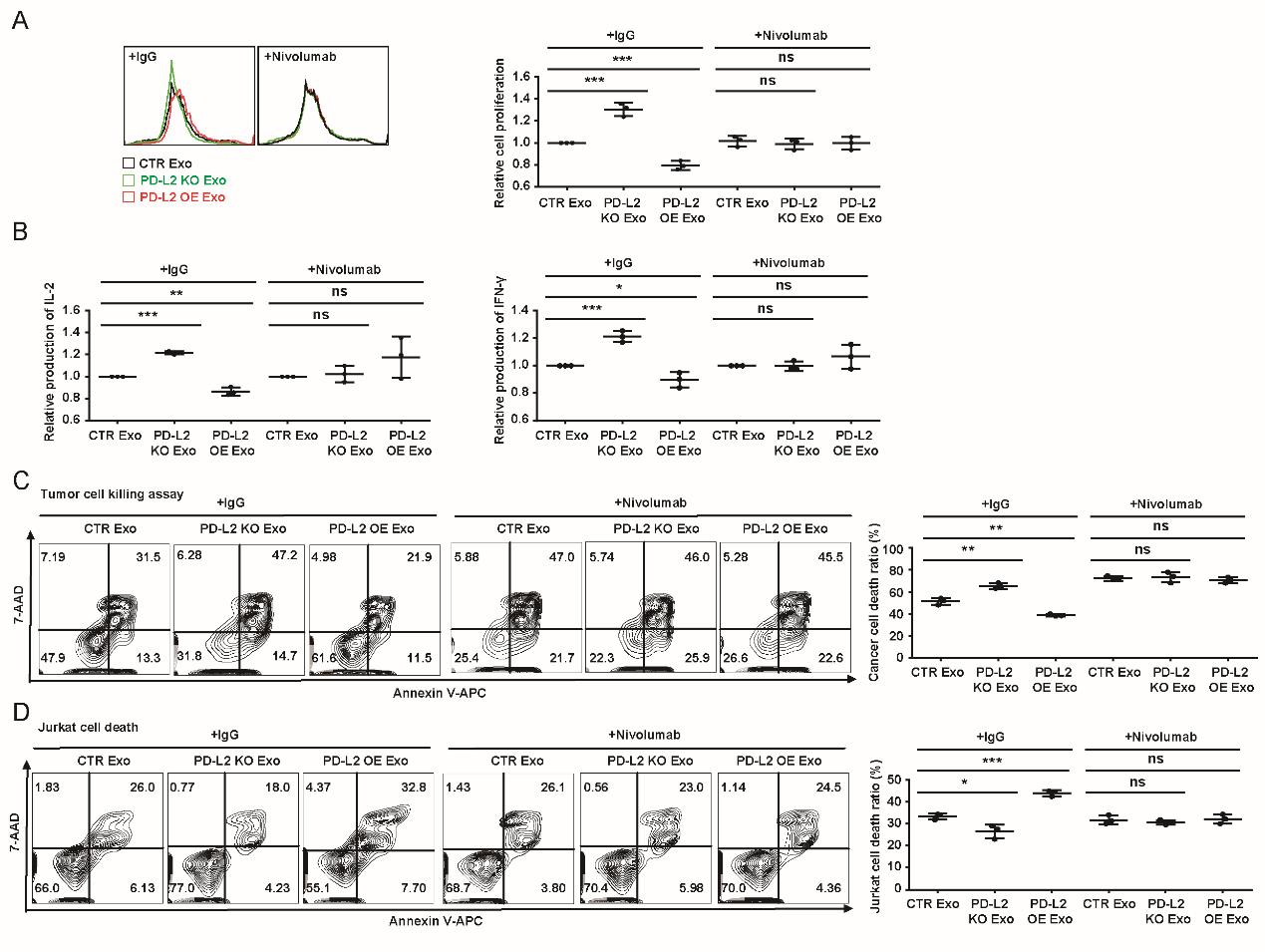


**Fig. S6 |** **TDE-PD-L2 Inhibits the Activity and Function of Jurkat via binding PD-1. Related to Figure 3.** (A) Representative FACS and quantification of Jurkat cell proliferation as 1/MFI treated with IgG or Nivolumab and the indicated exosomes. (B) Quantification of IL-2 (left) and IFN-γ (right) levels of the Jurkat cell treated with IgG or Nivolumab and the indicated exosomes. (C-D) Representative FACS and quantification for the dead cancer cells (C) and IgG or Nivolumab treated Jurkat cells (D) fed with the indicated exosomes. Data are presented as means ± SD, n=3. *p<0.05, **p<0.01, ***p<0.001, ns, not significant. unpaired t test.


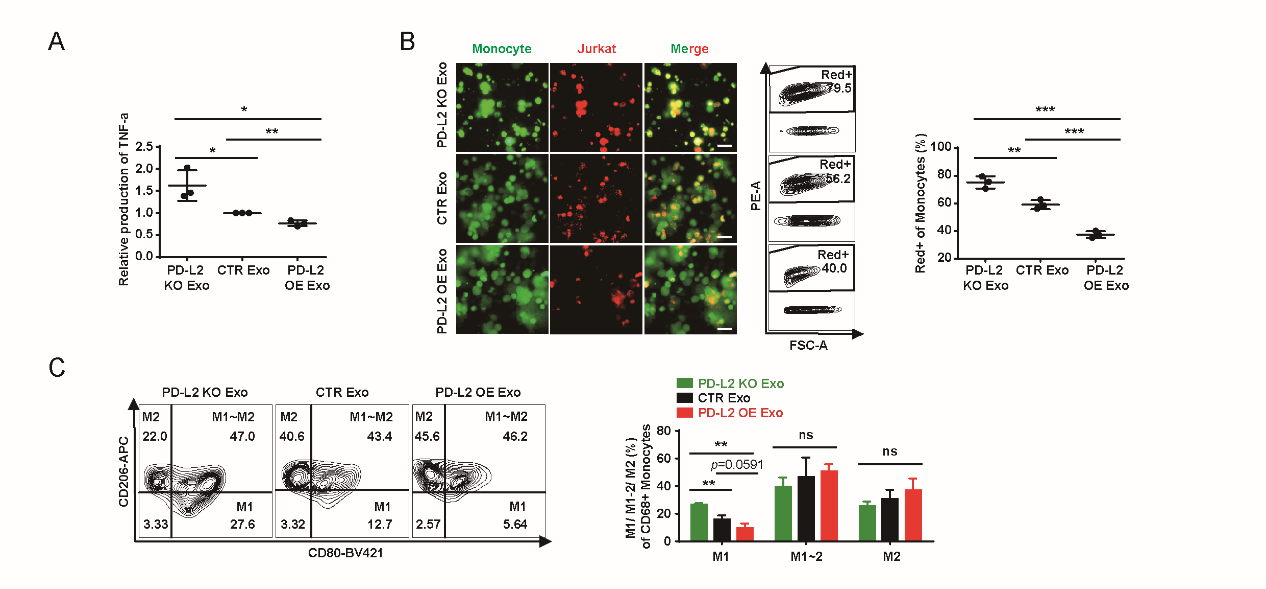


**Fig. S7 |** **TDE-PD-L2 Inhibits the Activity and Function of** **THP-1-derived macrophages.** (A) Quantification of TNF-α levels of the THP-1-derived macrophages treated with the indicated exosomes. (B) Macrophage phagocytic activity assay, representative images (left) of THP-1-derived macrophages (green) and Jurkat cells (red). Scale bars, 10µm. Representative FACS (middle) and quantification (right) of Jurkat-red+ in macrophages incubated with indicated exosomes. Evaluate macrophage phagocytic ability by the proportion of internally labeled Jurkat cells; a higher Red+% reflects enhanced phagocytic capacity. (C) Representative FACS and quantification of M1 (CD80+, CD206-), M1~M2 (CD80+, CD206+) and M2 (CD80-, CD206+) on CD68+ THP-1-derived macrophages fed with the indicated exosomes. Data are presented as means ± SD, n=3. *p<0.05, **p<0.01, ***p<0.001, ns, not significant. unpaired t test.


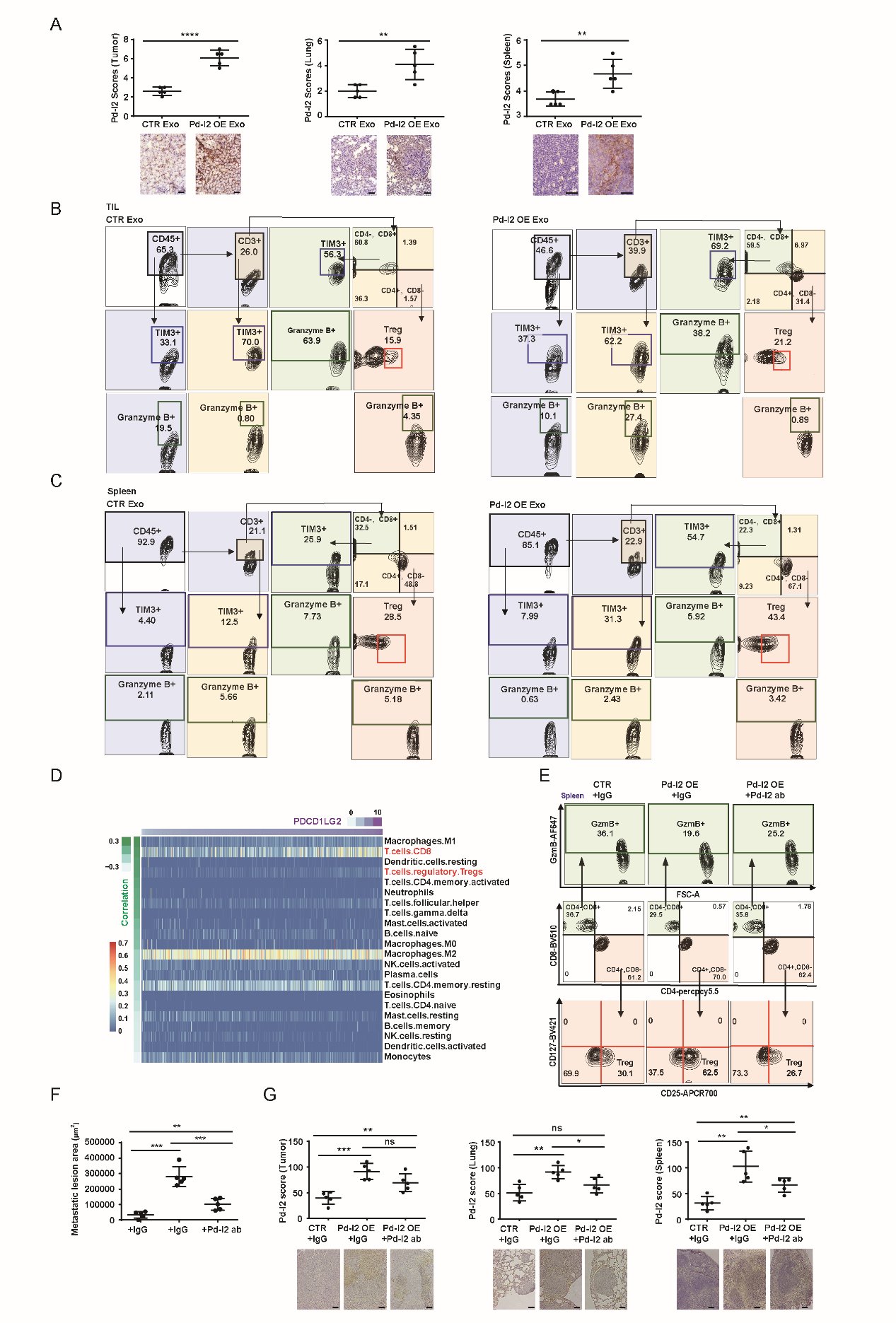
**Fig. S8 | TDE-PD-L2 systematic increase in Treg and decrease in CTL. Related to Figure 4.** (A) Quantification (top) and representative images (bottom) of PD-L2 levels in tumor of left kidney (left), lung (middle) and spleen (right) as determined by IHC analysis. Scale bars, 20 µm. (B-C) Representative FACS of the percentage of CD45^+^, CD3^+^, CD4^+^, CD8^+^, TIM3^+^, CD8^+^ cytotoxic T cells (CD8^+^, GzmB ^+^) and Treg (CD4^+^, CD25^+^, CD127^-^) in the TIL (B) or in the spleen cells (C). (D) Correlation between PD-L2 mRNA expression and imputed immune cells infiltration were analyzed using KIRC data from TCGA. (E) Representative FACS of CTL (top) and Treg (bottom) in spleen cells. Related to Figure 4-M. (F) Quantification of the area of metastatic lesions. (G) Quantification (top) and representative images (bottom) of PD-L2 levels of tumor of left kidney (left), lung (middle), spleen (right) of mice treated with indicated antibodies as determined by IHC analysis. Scale bars, 20 µm. Data are presented as means ±SD. *p<0.05, **p<0.01, ***p<0.001, ****p<0.0001, ns, not significant. unpaired t test.

**Supplementary Tables**

**Table S1: Reagent or source**

| **REAGENT or RESOURCE** | **SOURCE** | **IDENTIFIER** |
| --- | --- | --- |
| Antibodies |  |  |
| FITC Mouse Anti-Human CD3 | BD | Cat#555339 |
| FITC Isotype Mouse IgG1, kappa | BD | Cat#349041 |
| BV421 Mouse Anti-Human CD4 | BD | Cat#562842 |
| BV421 Isotype Mouse IgG1, kappa | BD | Cat#562438 |
| APC Isotype Mouse IgG1, kappa | BD | Cat#561421 |
| APC Mouse Anti-Human CD8 | BD | Cat#554681 |
| APC Mouse anti-Human CD279 (PD-1) | BD | Cat#558694 |
| APC Isotype Mouse IgG1, kappa | BD | Cat#555751 |
| Alexa Fluor 647 anti-Human/Mouse Granzyme B | BioLegend | Cat#515406 |
| Alexa Fluor 647 Isotype Mouse IgG1, kappa | BioLegend | Cat#400136 |
| BV786 Rat Anti-Mouse CD45 | BD | Cat#564225 |
| BV786 Isotype Rat IgG2b, kappa | BD | Cat#563334 |
| BV650 Hamster Anti-Mouse CD3e | BD | Cat#564378 |
| BV650 Isotype Hamster IgG1, kappa | BD | Cat#564388 |
| PerCP-Cy™5.5 Rat Anti-Mouse CD4 | BD | Cat#550954 |
| PerCP-Cy5.5 Isotype Rat IgG2a, kappa | BD | Cat#550765 |
| BV510 Rat Anti-Mouse CD8a | BD | Cat#563068 |
| BV510 Isotype Rat IgG2a, kappa | BD | Cat#562952 |
| APC-R700 Rat Anti-Mouse CD25 | BD | Cat#565134 |
| APC-R700 Isotype Rat IgG1, lambda | BD | Cat#565133 |
| BV421 Rat Anti-Mouse CD127 | BD | Cat#562959 |
| BV421 Isotype Rat IgG2b, kappa | BD | Cat#562603 |
| PE Mouse Anti-Mouse CD366 (TIM-3) | BD | Cat#566346 |
| PE Isotype Mouse IgG1, kappa | BD | Cat#554680 |
| PE anti-human CD68 Antibody | BioLegend | Cat#333807 |
| Brilliant Violet 421™ anti-human CD80 Antibody | BioLegend | Cat#305221 |
| APC anti-human CD206 (MMR) Antibody | BioLegend | Cat#321109 |
| PD-L2 Mouse Monoclonal Antibody (HIC, FACS) | UltraMAB | Cat#UM800115 |
| PD-L2 Mouse Monoclonal Antibody (WB) | OriGene | Cat#TA808990 |
| Mouse Anti-TSG101 antibody | abcam | Cat#ab83 |
| Anti-CD9 antibody | abcam | Cat#ab92726 |
| CD63 Rabbit monoclonal Antibody | abcam | Cat#ab134045 |
| CD69 anti-human Antibody | Biolegend | Cat#310902 |
| RAB27A Mouse monoclonal Antibody | abcam | Cat#ab55667 |
| Anti- Mouse CD4 antibody | abcam | Cat#ab183685 |
| GAPDH Antibody | proteintech | Cat#60004-1-Ig |
| Mouse IgG1, Kappa Monoclonal, Isotype Control | abcam | Cat#ab91353 |
| Rabbit IgG, monoclonal [EPR25A] - Isotype Control | abcam | Cat#ab172730 |
| Alexa Fluor 488 Goat anti-Mouse IgG (H+L), Adsorbed Secondary Antibody | Invitrogen | Cat#A-11029 |
| Alexa Fluor 555 Goat anti-Rabbit IgG (H+L), Adsorbed Secondary Antibody | Invitrogen | Cat#A-21429 |
| InVivoMAb anti-mouse PD-L2 | Bioxcell | Cat#BE0112 |
| InVivoPlus rat IgG2a isotype control | Bioxcell | Cat#BP0089 |
| Bacterial and virus strains |  |  |
| *Trans*5α Chemically Competent Cell | TransGen | Cat#CD201-01 |
| Biological samples |  |  |
| KIRC tissue microarray | Shanghai Outdo Biotech Company | Cat#HKidE150CS03 |
| KIRC tissue microarray | Shanghai Outdo Biotech Company | Cat#HKidE180Su03 |
| Chemicals, peptides, and recombinant proteins |  |  |
| Ionomycin, Free Acid, Streptomyces conglobatus in Solution | Sigma | Cat#407951 |
| PMA | Sigma | Cat#P1585 |
| CFSE | Thermo Fisher Scientific | Cat# C34554 |
| Fixable Viability Stain 620 | BD | Cat#565388 |
| Mouse CD16/CD32 Pure | BD | Cat#553141 |
| Rat/Ham Ig Kpa Comp Bead Set | BD | Cat#552845 |
| Ms Ig Kpa Comp Bead Set | BD | Cat#552843 |
| Recombinant Human IFN-γ Protein | RnD | Cat#285-IF-100 |
| Recombinant Human IL-2 Protein | RnD | Cat#BT-002-050 |
| Recombinant Human IL-4 Protein | RnD | Cat#204-IL-010 |
| Red Blood Cell Lysis Buffer | Solarbio | Cat#R1010-500ml |
| Taq Pro Universal SYBR qPCR Master Mix | Vazyme | Cat#Q712-02 |
| Invitrogen TRIzol | Thermo Fisher Scientific | Cat#15596026CN |
| Critical commercial assays |  |  |
| Human IFN-γ ELISA Kit | Biolegend | Cat#430104 |
| Human IL-2 ELISA Kit | Biolegend | Cat#431804 |
| Human TNF-a ELISA Kit | Biolegend | Cat#430215 |
| Human PD-L2 ELISA Kit | Thermo Fisher Scientific | Cat#BMS2215 |
| Human PD-L1 ELISA Kit | Thermo Fisher Scientific | Cat#BMS2212 |
| Mouse Tumor Infiltration Tissue Lymphocyte Separation Solution Kit | Solarbio | Cat#p9000 |
| Tumor Dissociation Kit, mouse | Miltenyi | Cat#130096730 |
| APC Annexin V Apoptosis Detection Kit with 7-AAD | biolegend | Cat#640930 |
| Genloci DNA extraction kit | Invitrogen | Cat#K182002 |
| gene knockout detection kit | Cruiser | Cat#GP0102 |
| Deposited data |  |  |
| PD-L2 KO exosomes feeded Lymphocytes RNA-seq | This Paper | GEO: GSE242798 |
| Lung cancer meRIP-Seq | Junho Choe et al.(2) | GEO: GSE117299 |
| Melanoma IGF2BP3 RIP-seq | Douglas Hanniford et al.(3) | GEO: GSE138710 |
| Experimental models: Cell lines |  |  |
| Mouse cell line: Renca | ATCC | Cat#CRL-2947 |
| Human cell line: Jurkat | ATCC | Cat#TIB-152 |
| Human cell line: THP-1 | ATCC | Cat#TIB-202 |
| Human cell line: HK2 | CBTCCC | Cat#GNHu47 |
| Human cell line: 786-O | CBTCCC | Cat#TCHu186 |
| Human cell line: 769-P | CBTCCC | Cat#TCHu215 |
| Human cell line: ACHN | CBTCCC | Cat#TCHu199 |
| Human cell line: Caki-1 | CBTCCC | Cat#TCHu135 |
| Human cell line: HEK293T | CBTCCC | Cat#GNHu 43 |
| Experimental models: Organisms/strains |  |  |
| Mouse: BALB/c | Beijing Vital River Laboratory Animal Technology Co. | Cat#211 |
| Mouse: BALB/c NOD SCID | Beijing Vital River Laboratory Animal Technology Co. | Cat#406 |
| Oligonucleotides |  |  |
| Primers for qRT-PCR, see Table S2 | This paper | N/A |
| shRNA/sgRNA/siRNA targeting sequence, see Table S2 | This paper | N/A |
| Recombinant DNA |  |  |
| pLVX-IRES-Neo | Clontech | Cat#632181 |
| pSIH1-H1-Puro | System Biosciences | Cat#SI500A-1 |
| pGK1.1(Puro)/CRISPR cas9 | Genloci | Cat#GP0132 |
| PMD2.G | Addgene | Cat#12259 |
| psPAX2 | Addgene | Cat#12260 |
| Software and algorithms |  |  |
| FlowJo-V10.6.2 | BD | https://www.flowjo.com/solutions/flowjo/downloads/previous-versions |
| GraphPad Prism-6 | GraphStats | https://www.graphpad-prism.cn/ |
| ImageJ-1.49V | Schneider et al.(4) | https://imagej.net/ |
| ImageScope-X64 | Leica Biosystems | https://www.leicabiosystems.com/zh-cn/digital-pathology/manage/aperio-imagescope/ |
| GSEA-4.0.0 | MSigDB | https://www.gsea-msigdb.org/gsea/index.jsp |
| R studio-4.2.3 | N/A | https://www.rstudio.com/ |
| CRISPR Design Tool | Feng Zhang Lab | https://zlab.bio/guide-design-resources |
| Other |  |  |
| Human Lymphocyte Separation tube | Dakewe Biotech | Cat#7121011 |
| 24 mm Transwell® with 0.4 µm Pore Polycarbonate Membrane Insert, Sterile | Corning | Cat#3412 |
| 6.5 mm Transwell® with 8.0 µm Pore Polycarbonate Membrane Insert, Sterile | Corning | Cat#3422 |
| Costar® 6 well plate, with lid flat bottom | Corning | Cat#3471 |
| Millex® 33mm PES .22um 250/pk RUO | Merck | Cat#SLGPR33RB |
| Ultracel-100 regenerated cellulose membrane, 4 mL sample volume | Merck | Cat#UFC810024 |
| Ultracel-100 regenerated cellulose membrane, 15 mL sample volume | Merck | Cat#UFC910024 |
| Ultracel® Regenerated Cellulose (30kDa MWCO), 15 mL sample volume | Merck | Cat#UFC903024 |
| MACS® SmartStrainers (70 µm) | miltenyi | Cat#130-098-462 |
| 0.22μm filter membrane | Merck | Cat#SLGP033NS |
| RPMI1640 | Gibco | Cat#11875119 |
| Fetal Bovine Serum | Gibco | Cat#10099158 |
| exosome-depleted FBS | Gibco | Cat#A27208-03 |
| DMEM/F-12 | Gibco | Cat#11330032 |
| Sodium Pyruvate | Gibco | Cat#11360070 |
| MEM Non-Essential Amino Acids Solution | Gibco | Cat#11140035 |
| DMEM | Gibco | Cat#11965092 |

**Table S2: Primers and target sequences**

| **qRT-PCR Primers** | | | | |
| --- | --- | --- | --- | --- |
| **Primer Name** | **Source** | | **Sequences** | |
| Human PD-L2-FW | This study | | CGCCTGGGACTACAAATATCTGA | |
| Human PD-L2-RV | This study | | GATCCTGAGGAAATGAGTGTTTATTTT | |
| Human PD-1-FW | This study | | TCGTGCTAAACTGGTACCGC | |
| Human PD-1-RV | This study | | CTGACCACGCTCATGTGGAA | |
| Human GAPDH-FW | This study | | CAGGGCTGCTTTTAACTCTGGTA | |
| Human GAPDH-RV | This study | | CATGGGTGGAATCATATTGGAAC | |
| Human Rab27a-FW | This study | | GTTGATGGAGCGAACTGCTTTT | |
| Human Rab27a-RV | This study | | CAGGGTAGAGAACCGCTTGTTAT | |
| Mice Pd-l2-FW | This study | | TACAGGGGAGGTGCAGCTTA | |
| Mice Pd-l2-RV | This study | | CGTTCTGGGGACTTTGGGTT | |
| Mice Gapdh-FW | This study | | CATCACTGCCACCCAGAAGACTG | |
| Mice Gapdh-RV | This study | | ATGCCAGTGAGCTTCCCGTTCAG | |
| **shRNA/sgRNA for KD/KO** | | | | |
| **Name** | | **Source** | | **Sequences-Forwards** |
| Human Rab27a-sh1 | | This study | | GCTGCCAATGGGACAAACATA |
| Human Rab27a-sh2 | | This study | | GCGTTCTTCAGAGATGCTATG |
| Human PD-L1-sg1 | | This study | | GTCCAGATGACTTCGGCCTTGGG |
| Human PD-L1-sg2 | | This study | | TGGTGGTGGTCTTACCACTCAGG |
| Human PD-L1-sg3 | | This study | | ACAGCTGAATTGGTCATCCCAGG |
| Human PD-L2-sg1 | | This study | | AAGTGGCTCTTTCACGGTGTGGG |
| Human PD-L2-sg2 | | This study | | TAATCATCTATGGGGTCGCCTGG |
| Human PD-L2-sg3 | | This study | | GACTTGAGGTATGTGGAACGAGG |

**Reference:**

1. Minciacchi VR, You S, Spinelli C, Morley S, Zandian M, Aspuria PJ, et al. Large oncosomes contain distinct protein cargo and represent a separate functional class of tumor-derived extracellular vesicles. Oncotarget. 2015;6(13):11327-41.

2. Choe J, Lin S, Zhang W, Liu Q, Wang L, Ramirez-Moya J, et al. mRNA circularization by METTL3-eIF3h enhances translation and promotes oncogenesis. Nature. 2018;561(7724):556-60.

3. Hanniford D, Ulloa-Morales A, Karz A, Berzoti-Coelho MG, Moubarak RS, Sanchez-Sendra B, et al. Epigenetic Silencing of CDR1as Drives IGF2BP3-Mediated Melanoma Invasion and Metastasis. Cancer cell. 2020;37(1):55-70 e15.

4. Schneider CA, Rasband WS, Eliceiri KW. NIH Image to ImageJ: 25 years of image analysis. Nat Methods. 2012;9(7):671-5.
