# Supplementary figures and images for: PD-L2 of tumor-derived exosomes mediates the immune escape of cancer cells via the impaired T cell function
Source: Cell Death Dis. 2024 Nov 7;15(11):800. doi: 10.1038/s41419-024-07191-7 (PMC11544247; doi:10.1038/s41419-024-07191-7)

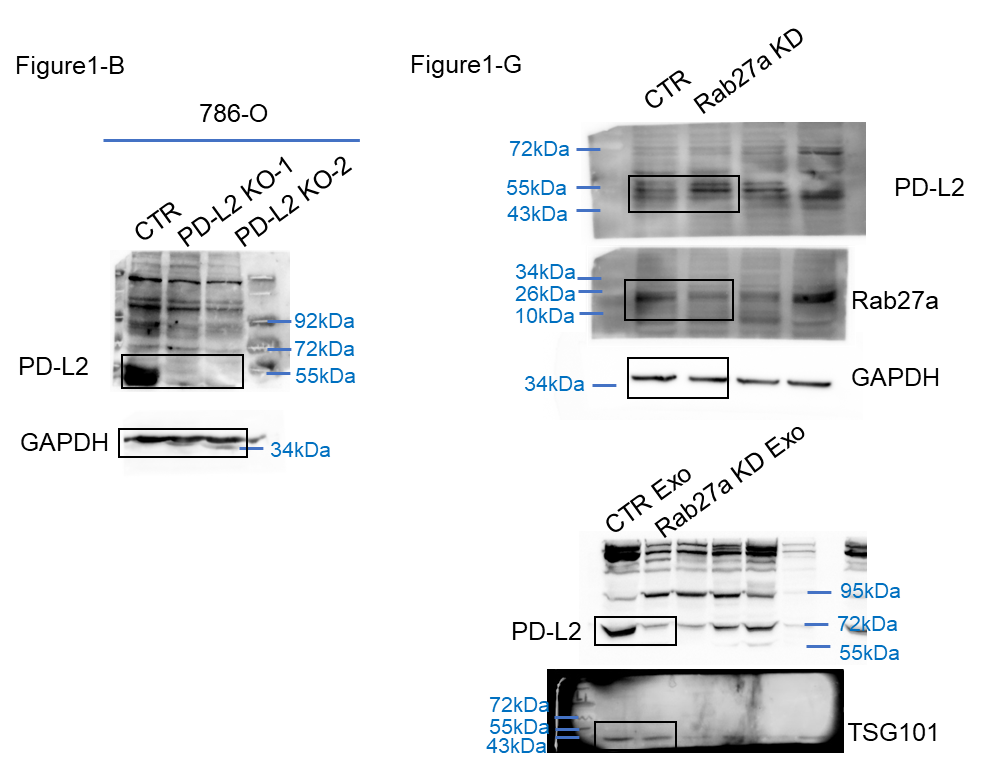

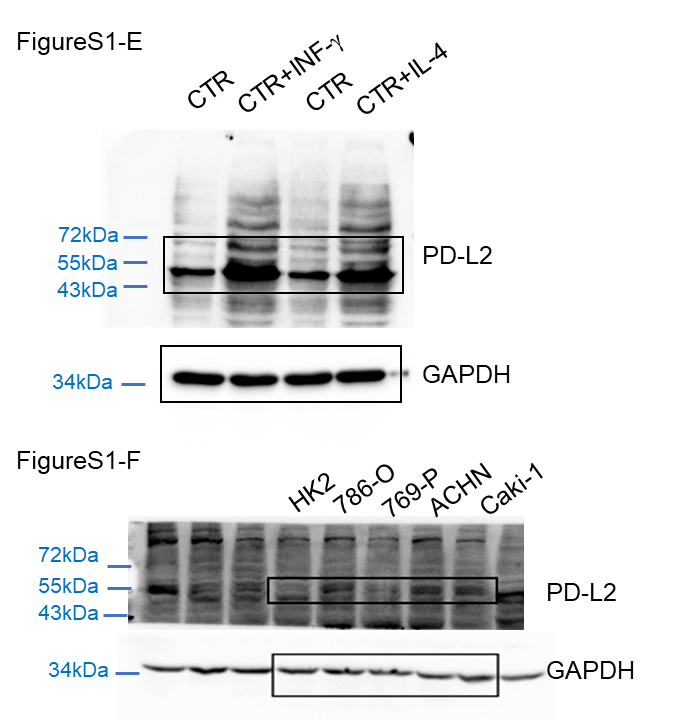


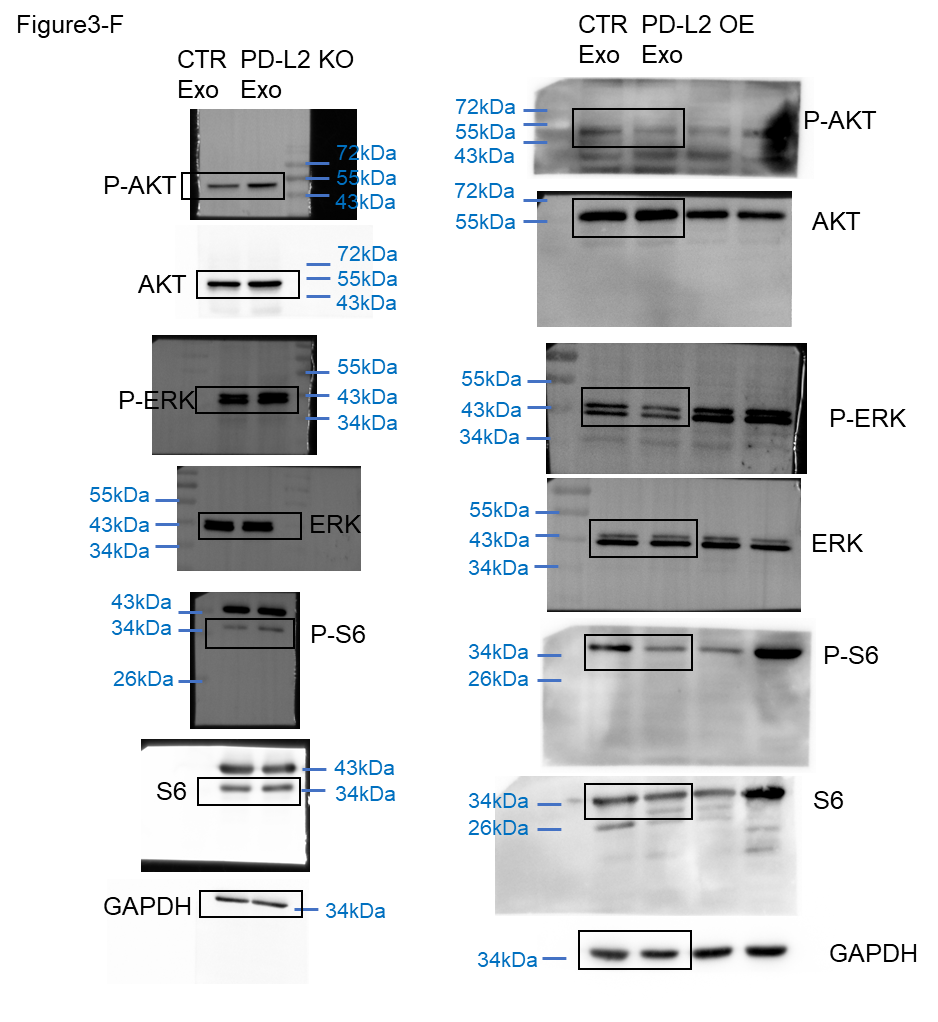

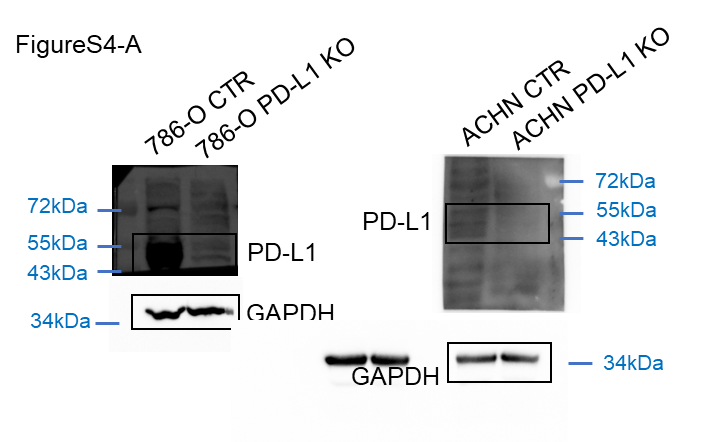

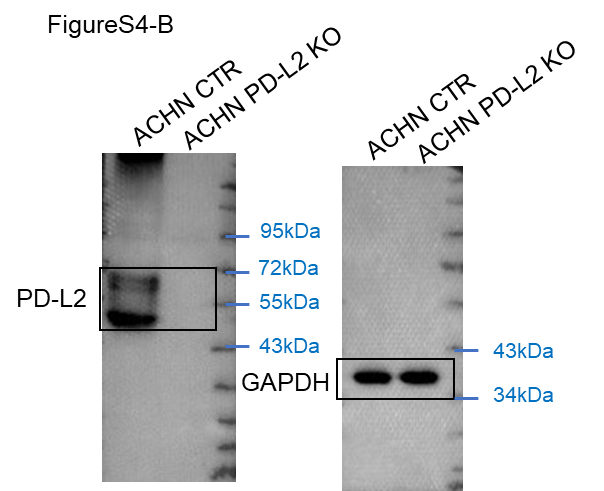

Supplement: Supplementary file 2 — original western blots [file 41419_2024_7191_MOESM2_ESM.docx]
